# Comprehensive analysis of the prognosis and immune infiltration landscape of RNA methylation-related subtypes in pancreatic cancer

**DOI:** 10.1186/s12885-022-09863-z

**Published:** 2022-07-21

**Authors:** Siyuan Lu, Jie Hua, Jiang Liu, Miaoyan Wei, Chen Liang, Qingcai Meng, Bo Zhang, Xianjun Yu, Wei Wang, Jin Xu

**Affiliations:** 1 Department of Pancreatic Surgery, Fudan University Shanghai Cancer Center, No. 270 Dong’An Road, Shanghai, 200032 China; 2 Department of Oncology, Shanghai Medical College, Fudan University, Shanghai, China; 3 Shanghai Pancreatic Cancer Institute, No. 270 Dong’An Road, Shanghai, 200032 China; 4 Pancreatic Cancer Institute, Fudan University, Shanghai, China

**Keywords:** Pancreatic cancer, RNA methylation, Bioinformatics analysis, Immune infiltration

## Abstract

**Background:**

RNA methylation refers to a form of methyl modification in RNA that modulates various epigenetic alterations. Mounting studies have focused on its potential mechanisms in cancer initiation and progression. However, the prognostic value and potential role of RNA methylation in the immune microenvironment of pancreatic cancer remain unclear.

**Methods:**

Comprehensive bioinformatics analysis was performed to illuminate the expression profiles of RNA methylation modulators. In addition, the ConsensusClusterPlus algorithm was utilized to identify two remarkably different subtypes, and a feasible risk stratification method was established to accurately estimate prognosis. In addition, we validated our signature at the cytology and histology levels and conducted functional experiments to explore the biological functions of our key genes.

**Results:**

Two subtypes with remarkable survival differences were identified by the consensus clustering algorithm. Cluster 2 tended to have higher expression levels of RNA methylation regulators and to be the high RNA methylation group. In addition, cluster 1 exhibited a significantly higher abundance of almost all immune cells and increased immune checkpoint expression compared to cluster 2. Chemotherapeutic sensitivity analysis indicated that there were significant differences in the sensitivity of four of the six drugs between different subgroups. Mutation investigation revealed a higher mutation burden and a higher number of mutations in cluster 2. An accurate and feasible risk stratification method was established based on the expression of key genes of each subtype. Patients with low risk scores exhibited longer survival times in one training (TCGA) and two validation cohorts (ICGC, GSE57495), with *p* values of 0.001, 0.0081, and 0.0042, respectively. In addition, our signature was further validated in a cohort from Fudan University Shanghai Cancer Center. The low-risk group exhibited higher immune cell abundance and immune checkpoint levels than the high-risk group. The characteristics of the low-risk group were consistent with those of cluster 1: higher stromal score, estimate score, and immune score and lower tumor purity. Additionally, cell function investigations suggested that knockdown of CDKN3 remarkably inhibited the proliferation and migration of pancreatic cancer cells.

**Conclusions:**

RNA methylation has a close correlation with prognosis, immune infiltration and therapy in pancreatic cancer. Our subtypes and risk stratification method can accurately predict prognosis and the efficacy of immune therapy and chemotherapy.

**Supplementary Information:**

The online version contains supplementary material available at 10.1186/s12885-022-09863-z.

## Introduction

Pancreatic cancer is regarded as one of the most aggressive and lethal malignances and has an extremely high mortality rate [1]. Given the lack of early symptoms and late diagnosis, patients with pancreatic cancer always miss the opportunity to undergo radical surgery [2]. Drug resistance makes the effects of various common chemotherapy regimens for pancreatic cancer unsatisfactory [3]. Additionally, newly emerging immunotherapy drugs have achieved disappointing outcomes in the treatment of pancreatic cancer, which may be attributed to the cold immune microenvironment of pancreatic cancer [4]. The immune microenvironment of pancreatic cancer tend to be less infiltrated by immune cells, leading to the low reactivity of pancreatic cancer to immunotherapy [4] Therefore, new risk stratification methods and biomarkers are urgently needed for clinical practice and therapy in pancreatic cancer.

RNA methylation is a form of RNA methyl modification that modulates epigenetic alterations [5]. More than 70 types of RNA methylation modifications have been identified in messenger RNA (mRNA) and noncoding RNA (ncRNA), and the most essential types in humans include N6-methyladenosine (m6A), 5-methylcytosine (m5C), and 7-methylguanosine (m7G) modification [5–7]. m6A methylation refers to adenosine methylation at the N6 position; the form of methylation was discovered in 1974 and thought to be the most common [8, 9]. m5C methylation adds a methyl group to cytosine at the 5th carbon in mRNAs [10], enhancer RNAs (eRNAs) [11], transfer RNAs (tRNAs) [12], small RNAs (sRNAs) [13], and ribosomal RNAs (rRNAs) [13]. m7G methylation modifies the N7 guanosine with a methyl group and has been found to exist in various mRNAs and ncRNAs [14, 15]. RNA methylation is a reversible process requiring various auxiliary enzymes and binding proteins, including methyltransferases, demethylases, and modified RNA binding proteins known as “writers”, “erasers”, and “readers”, respectively. Writers dominate the RNA methylation process [5]. In the writer complex consisting of METTL3 and METTL14, METTL3 functions as the catalytic subunit, while METTL14 facilitates RNA binding. In addition, the zinc finger protein ZC3H13 has also been reported to promote the nuclear localization of the writer complex [16]. Erasers play an essential role in RNA demethylation. FTO and ALKBH5 are key erasers in demethylation. FTO is mainly localized to the nucleus and mediates m6A demethylation. In addition, FTO can also promote m6A demethylation without affecting transcriptional stability [17]. Correspondingly, readers recognize and bind methylated RNA, affecting its posttranscriptional modification [18] The reader YTHDC1 regulates mRNA splicing and alters transcript metabolism [19], and YTHDC2 has also been found to bind methylation sites and mediate mRNA stability [20].

Many studies have demonstrated the potential connection between RNA methylation and various biological processes, especially the occurrence and progression of malignant tumors [21, 22]. Wang et al. revealed that elevated expression of METTL3 (m6A writer) contributed to the progression and metastasis of gastric cancer and indicated poor prognosis [23]. Interestingly, METTL14 (m6A writer) was reported to exhibit decreased levels in colorectal cancer and to attenuate colorectal cancer cell invasion and proliferation by suppressing m6A modification [24]. In addition, pancreatic cancer was also found to have upregulated METTL3 and downregulated ALKBH5 (m6A eraser) expression, and this dysregulation significantly influenced RNA methylation and resulted in poor clinical outcomes. METTL3 mainly affects RNA splicing and cellular regulation to promote chemotherapy and radiation tolerance, while ALKBH5 mainly regulates WIF-1 RNA methylation and Wnt signaling to modulate tumor progression [25, 26]. In conclusion, the disturbance of RNA methylation is closely related to the occurrence and progression of different tumors.

Many scientists have shed light on the correlation between RNA methylation and the tumor immune microenvironment [27]. Some studies have indicated that T-cell differentiation is regulated by METTL3 because it initiates the methylation of IL7-related pathways. Depletion of YTHDF1 (m6A reader) can also suspend the related methylation process and enhance the antigen presentation competence of dendritic cells, ultimately affecting the activation of other immune cells [28]. Additionally, RNA methylation may also be involved in innate immunity modulation. Gu et al. revealed that deletion of FTO (m6A eraser) contributed to the inhibition of M1 and M2 macrophages by silencing STAT1 and NF-κB pathway activity [29]. Given the potential correlation between RNA methylation and the tumor immune microenvironment of pancreatic cancer, it may be promising to analyze and investigate biological targets and therapeutic methods for immunotherapy of pancreatic cancer through RNA methylation.

In our research, we comprehensively analyzed the potential relationship of RNA methylation with distinct pancreatic cancer subtypes and the role of RNA methylation in risk classification, prognostic assessment, immune regulation and treatment.

## Materials and methods

### Dataset acquisition and RNA methylation modulator identification

We extracted the sequencing, mutation (version varscan2), and clinical data of pancreatic cancer patients from the UCSC Xena database (https://xena.ucsc.edu/). A total of 124 complete data points was retained after excluding those from benign tumor and neuroendocrine cancer samples (GDC TCGA PAAD, log2(tpm + 1) values). In addition, the transcriptome data of PACA-AU (80 samples) and GSE57495 (63 samples) were obtained from the ICGC (https://dcc.icgc.org/) and GEO databases, respectively (https://www.ncbi.nlm.nih.gov/geo/). Overall, 38 critical RNA methylation modulators (readers, writers, or erasers of RNA methylation) were identified from previously published literature, and the information is listed in Table 1. The transcriptome data of cancer and normal pancreatic samples (TCGA TARGET GTEx cohort, log2(tpm + 0.001) values) were obtained from the UCSC Xena database and utilized to analyze the expression profiles of RNA methylation modulators.

**Table 1:** List of RNA methylation regulators of M5C, M6A, and M7G methylation

	Writer	Reader	Eraser
M5C	TRDMT1 [30], NSUN1 [31], NSUN2 [31] NSUN3 [31], NSUN4 [31], NSUN5 [31] NSUN6 [31], NSUN7 [31], DNMT1 [32] DNMT2 [33], DNMT3A [32], DNMT3B [32]	ALYREF [34], YBX1 [34]	TET2 [35]
M6A	METTL3 [36], METTL14 [36], METTL16 [37], WTAP [38], KIAA1429 [39], RBM15 [40], RBM15B [41], ZC3H13 [42]	EIF3A [43], IGF2BP1 [44], IGF2BP2 [44], IGF2BP3 [44], YTHDC1 [45], YTHDC2 [45], YTHDF1 [45], YTHDF2 [46], YTHDF3 [47], HNRNPC [48], HNRPA2B1 [44]	FTO [49], ALKBH5 [50]
M7G	METTL1 [51], WDR4 [52]		

### Identification of significantly different subtypes based on expression patterns of RNA methylation modulators

A consensus clustering algorithm was employed to identify optimal subtypes based on a matrix composed of RNA methylation regulators using the R package “ConsensusClusterPlus” (K-means, Euclidean distance, reps = 1000, pItem = 0.8, clusterAlg = “hc”) [53]. Additionally, we performed survival analysis to compare the prognoses of different subtypes (R package “survminer 0.4.9”, R package “survival 3.3.7”, conf.int = T, pval = T, risk.table = T) [54, 55].

### Elucidating the immune characteristics, chemotherapy sensitivity and mutation landscape of different subtypes

We compared the immune infiltration, chemotherapy sensitivity and mutation landscapes of the subtypes. The R package “GSVA” (version 1.38.2, mx.diff = FALSE, verbose = FALSE, parallel.sz = 1) [56] and the CIBERSORT algorithm (version 1.0.3, perm = 100, QN = TRUE) [57] were used to estimate the proportions of infiltrating immune cells in various subtypes. The R package “PRRophetic” (version 0.5, batchCorrect = ‘eb’, powerTransformPhenotype = T, removeLowVaryingGenes = 0.2, minNumSamples = 10) was used to estimate the common drug IC50 values of various samples [58]. Based on the drug sensitivity data from the Cancer Genome Project, users could predict the IC50 value from sample expression data. The mutation landscapes of different subtypes were also evaluated by the R package “maftools” (version 2.6.05, rmOutlier = TRUE, addStat = ‘median’, dashboard = TRUE, titvRaw = FALSE) [59]. The differences in immune cell abundance, drug IC50, and mutation burden between different risk groups were compared through the Wilcoxon test. The results were visualized with box plots and violin plots using the R package “ggpubr” (version 0.4.0) [60] and “ggplot2” (version 3.3.5), respectively [61].

### Differential expression and coexpression analyses

The empirical bayesian algorithm was employed by the R package “limma” to identify RNA methylation-related differentially expressed genes (DEGs) (version 3.46.0, abs (logFC) > 1, P.value < 0.05) [62]. A volcano plot of the DEGs was drawn by the R package “ggplot2” (version 3.3.5) [61]. In addition, we analyzed the correlations among the DEGs, and a correlation heatmap was utilized to visualize the coexpression profiles. The coexpression analysis was performed and the heatmap was generated by the R package “corrplot” (version 0.92, method = “circle”,insig = “pch”, number.cex = 0.5, order = “AOE”) [63]. Functional analysis, including Gene Ontology (GO) and Kyoto Encyclopedia of Genes and Genomes (KEGG) analyses and gene set enrichment analysis (GSEA), was performed by the R packages “clusterProfiler 3.18.1” (pvalueCutoff =0.05, qvalueCutoff =0.05, readable = TRUE) [64] and “org. Hs.eg.db 3.12.0” (fromType = “SYMBOL”, toType = “ENTREZID”) [65].

### Identification of RNA methylation-related hub genes and construction of a risk model

We screened the DEGs to identify the most important biomarkers. The filtering methods included univariate Cox regression analysis, LASSO regression analysis, random forest algorithm analysis, and multivariate Cox regression analysis. Univariate Cox regression analysis was used to identify survival-related genes (R package “survminer 0.4.9”, R package “survival 3.3.7”) [54, 55]. Significant genes (*P* value < 0.005) identified in the univariate Cox regression analysis were included in the LASSO regression and random forest analyses. We then combined the hub genes identified in the LASSO regression and random forest analyses. The random forest algorithm analysis was performed with the R package “randomForestSRC” (version 2.12.0, set.seed = 60, ntree = 100, nsplit = 1) [66]. LASSO regression analysis was performed with the R package “glmnet” (version 4.1.2, nfold = 1000, family = ‘cox’) [67]. Multivariate Cox regression analysis was used to eliminate collinearity and construct a risk signature (R package “survival 3.3.7”) [55]. The risk score formula was as follows: ${\sum}_1^n coef\ast \exp \left(\mathrm{xn}\right)$. The R packages “survival” (version 3.3.7) and “surviminer” (version 0.4.8) were used to draw survival curves and compare the prognosis of different risk groups [54, 55]. Additionally, we established a clinical nomogram integrating the risk signature and some clinical characteristics to increase clinical applicability (R package “rms 6.2.0”) [68]. Area under the curve (AUC) analysis was used to evaluate the risk signature, and the receiver operating characteristic (ROC) curves were drawn by the R package “timeROC” (version 0.4.0, marker = lpFit, cause = 1, weighting = “marginal”,ROC = T, iid = T) [69].

### Comprehensively analyzed our risk model in immune regulation activities, immune checkpoint therapy and chemotherapy

The CIBERSORT [57] and ssGSEA [70] algorithms (method = ‘ssgsea’, kcdf = ‘Gaussian’, abs.ranking = TRUE) were used to estimate the immune cell infiltration between various risk groups. The R package “ESTIMATE 1.0.13” was used to calculate the stromal score, estimate score, immune score, and tumor purity of all samples [71]. These algorithms allowed us to assess various immune cell scores and proportions based on the sample expression matrix. In addition, we also compared the expression levels of immune checkpoints and the IC50 values of chemotherapeutic drugs in different risk groups. Information on immune checkpoints is presented in Table S1. The R package ‘PRRophetic’ (version 0.5, batchCorrect = ‘eb’, powerTransformPhenotype = T, removeLowVaryingGenes = 0.2, minNumSamples = 10) was used to estimate the common drug IC50 values of various samples [58]. The Wilcoxon test was used to compare the differences in immune checkpoints and chemotherapeutic drug IC50 values between different risk groups, and a *p* value < 0.05 was considered significant.

### Comparison of our risk model with other published risk signatures

To prove the superiority of our risk model in prognostic ability, several published models were compared with our model in the following aspects: survival curve, ROC curve and c-index (“rms” 6.2.0) [68]. We mainly compared our model with three published models, the 3-gene model of Tang et al [72], the 4-gene model of Meng et al [73], and the 5-gene model of Xu et al [74]. A well-differentiated survival curve, a better ROC curve area and a larger c-index indicate more ideal prediction performance.

### Cell culture and qRT–PCR verification

We obtained one human normal pancreatic epithelial cell line (HPDE) and four human pancreatic cancer cell lines (SW 1990, BxPC-3, CFPAC-1, PANC-1) to verify the expression levels of ANLN, ARNTL2, CDKN3, and FAM53B. In addition, the expression levels of all RNA methylation regulators in different cell lines were also measured and compared. All cell lines were cultured in Dulbecco’s modified Eagle’s medium (DMEM) with 10% fetal bovine serum (FBS) and 1% antifungal. Additionally, 70 clinical samples with complete follow-up information that underwent pancreatic cancer resection were extracted from Fudan University Shanghai Cancer Center. We extracted the RNA of these paraffin-embedded specimens to verify the clinical application value of our risk model. The primer sequences of all genes are provided in Table S2. The expression data were normalized to GAPDH, and the relative mRNA expression level was calculated by the 2^-ΔΔCt^ method.

### Flow cytometry

PANC-1 cells were chosen to perform the flow cytometry experiment. A PE Annexin V Apoptosis Detection Kit (BD, cat: 559763) was used to stain PANC-1 cells, and a FACSCalibur flow cytometer was used to count them.

### Transwell migration assay

To detect the migration ability, PANC-1 cells (5 × 10^4^ cells) were cultured in the upper chamber with 200 μL of serum-free medium. Then, 500 μl medium with 20% FBS was added to the lower chamber. After fixing, staining and washing the upper chamber cells, we counted the migrating cells with 5 random areas per chamber.

### EdU assay

PANC-1 cells (3 × 10^4^ cells) were cultured in 96-well plates with 4 replicate wells and incubated with EdU working solution for two hours (BeyoClick™ EdU Cell Proliferation Kit with Alexa Fluor 594, China). After fixation, membrane rupture and nuclear staining, we used a fluorescence microscope (OLYMPUS, Tokyo, Japan) to capture fluorescence images.

## Results

### Expression profiles of RNA methylation modulators in pancreatic cancer

**Fig. 1:**
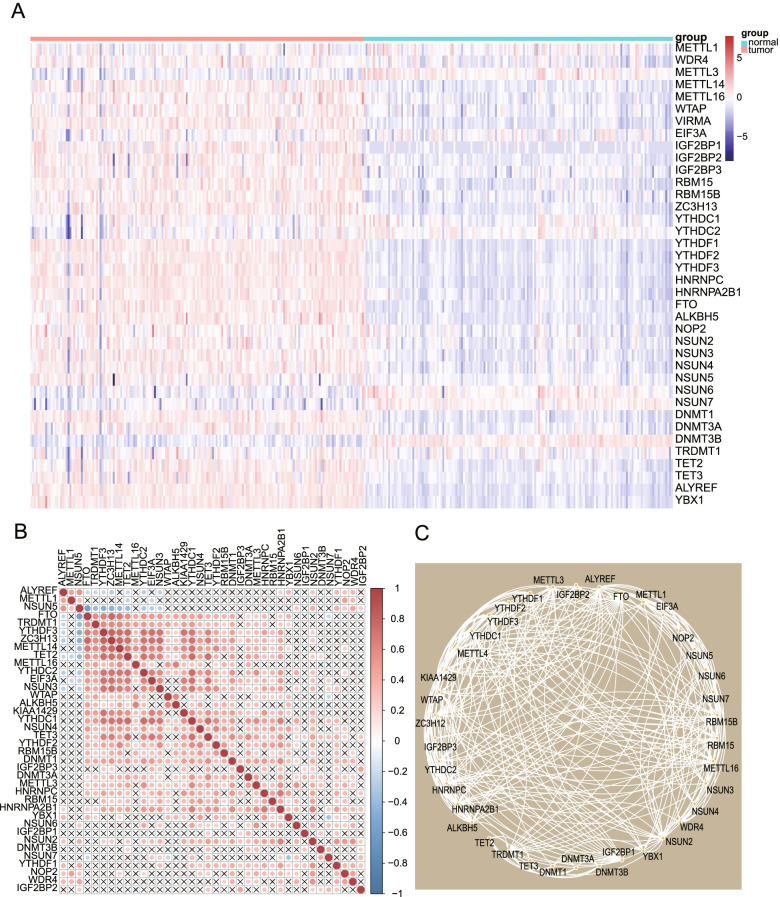
Expression profiles of RNA methylation modulators in pancreatic cancer. **A** The heatmap shows the expression profiles of RNA methylation modulators in normal tissues and cancer tissues. **B** Coexpression analysis indicated strong correlations among the majority of genes. Red indicates a positive correlation, blue indicates a negative correlation, and “×” means that the *p* value of the correlation was > 0.05. **C** Protein network demonstrating the correlation among RNA methylation modulators

To investigate the potential role of RNA methylation modulators (including m5C, m6A, and m7G modulators) in the occurrence and progression of pancreatic cancer, we compared the expression profiles of 38 RNA methylation modulators between pancreatic cancer samples and normal samples. The heatmap shows the expression levels of these 38 RNA methylation modulators (Fig. 1A). The box plot further shows a comparison of tumor and normal samples (Fig. S1A). Almost all RNA methylation modulators showed elevated expression levels in tumors compared to normal tissue (except METTL1, EIF3A, YTHDC1, and YTHDC2) (Fig. S1A). In addition, NSUN6, NSUN7, and DNMT3B exhibited downregulated trends in pancreatic cancer (Fig. S1A). Coexpression analysis indicated high correlation among the majority of RNA methylation modulators (Fig. 1B). We further explored direct associations between these regulators at the proteomic level. The protein interaction network further demonstrates the strong correlations among these RNA methylation modulators (Fig. 1C).

### Identification of two clusters of pancreatic cancer with different survival characteristics

**Fig. 2:**
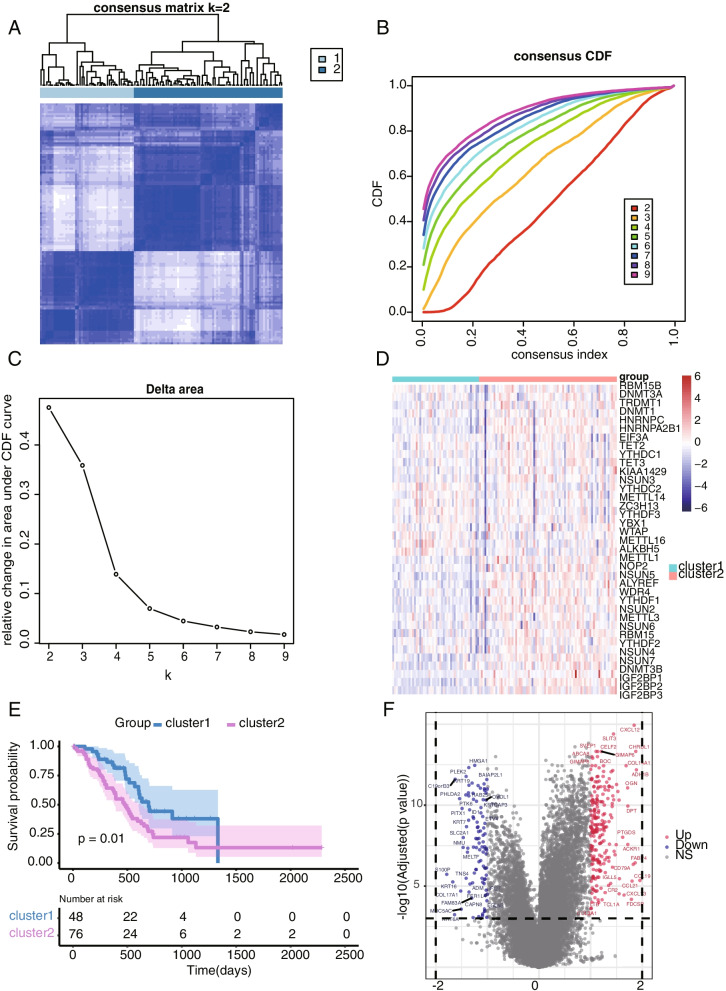
Two clusters of pancreatic cancer samples with different survival characteristics. **A-C** k = 2 exhibited the best clustering performance with the smallest CDF value and the largest CDF area change. **D** The heatmap shows the expression level of RNA methylation modulators in cluster 1 and cluster 2. **E** Cluster 1 had better overall survival than cluster 2. **F** The volcano plot depicts the differentially expressed genes between the two clusters. Red represents upregulated differentially expressed genes, blue represents downregulated differentially expressed genes, and gray represents nonsignificant differentially expressed genes. A *P* value < 0.05 was considered significant

We identified 2 unique expression patterns through unsupervised clustering: cluster 1 and cluster 2 (Fig. 2A, B, C). A heatmap was generated to show the expression levels of the RNA methylation modulators. The expression level of the RNA methylation modulators in cluster 2 was higher than that in cluster 1, indicating that cluster 2 featured high RNA methylation (Fig. 2D). Furthermore, we compared prognoses between cluster 1 and cluster 2, and cluster 1 exhibited better overall survival (*P* value =0.01) (Fig. 2E). A total of 2130 DEGs were identified between the two clusters by the R package “limma” (*p* value*<* 0.05). The volcano plot depicts the upregulated and downregulated DEGs, which are marked in red and blue, respectively (Fig. 2F).

### Patients in the two clusters differed in immune landscape, drug sensitivity, and mutation burden

**Fig. 3:**
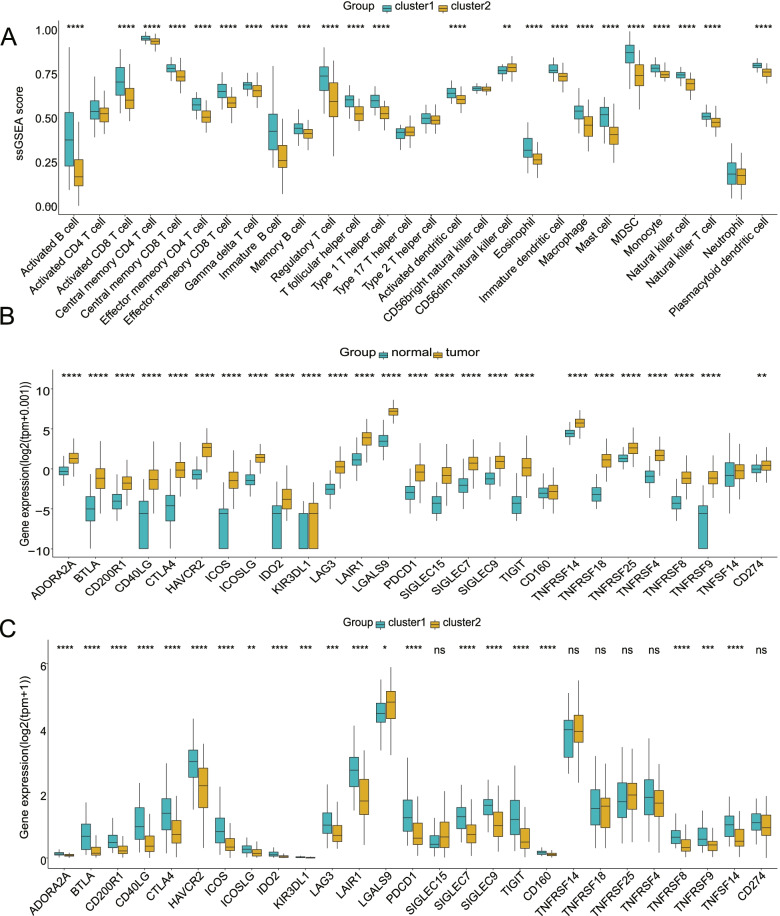
Patients in the two clusters differed in immune landscape. **A** Cluster 1 exhibited a higher abundance of almost all immune cells than cluster 2 (ssGSEA). (Comparisons were made by the Wilcoxon test, the lines in the boxes represent medians, and the asterisks represent *p* values. *P < 0.05, **P < 0.01, ***P < 0.001, ****P < 0.0001, ns *P* > 0.05.) **B** Pancreatic cancer samples showed elevated immune checkpoint levels compared to normal samples. (Comparisons were made by the Wilcoxon test, the lines in the boxes represent medians, and the asterisks represent p values. *P < 0.05, **P < 0.01, ***P < 0.001, ****P < 0.0001, ns P > 0.05.) **C** Cluster 1 exhibited relatively higher expression levels of almost all immune checkpoints than cluster 2. (Comparisons were made by the Wilcoxon test, the lines in the boxes represent medians, and the asterisks represent p values. *P < 0.05, **P < 0.01, ***P < 0.001, ****P < 0.0001, ns P > 0.05)

**Fig. 4:**
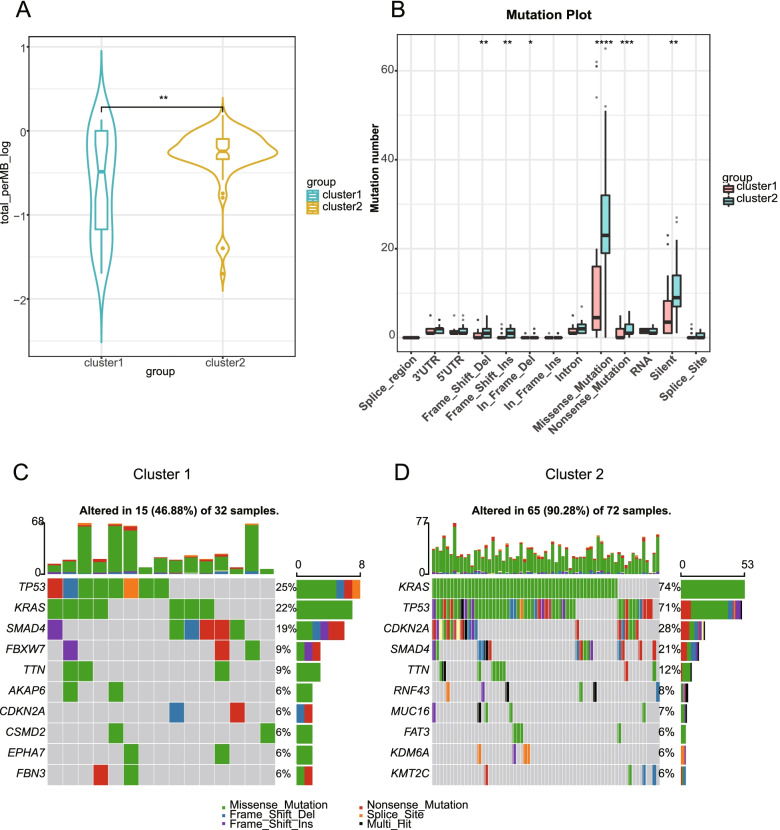
Patients in the two clusters differed in mutation landscape. **A** Comparison of the TMB score between cluster 1 and cluster 2 (Wilcoxon test; the asterisks represent p values; **P < 0.01) **B** Comparison of mutation types between cluster 1 and cluster 2 (Wilcoxon test; the lines in the boxes represent medians, and the asterisks represent p values; *P < 0.05, **P < 0.01, ***P < 0.001, ****P < 0.0001, ns P > 0.05.) **C**, **D** Waterfall plot depicting the mutated genes of patients in different clusters

To further investigate the practical application utility of the clusters, we assessed immune and mutation characteristics and drug sensitivity in the two clusters. Cluster 1 exhibited a significantly higher abundance of almost all immune cells than cluster 2 (Fig. 3A, ssGSEA), except for activated NK cells and M0 macrophages (Fig. S1B, CIBERSORT). Pancreatic cancer samples showed elevated immune checkpoint expression levels compared to normal samples, indicating changes in the immune microenvironment (Fig. 3B). Interestingly, cluster 1 exhibited relatively higher expression of almost all immune checkpoints than cluster 2, except for LGALS9, indicating that it may have high responsiveness to immune checkpoint inhibitors (Fig. 3C). However, in terms of drug resistance, the different clusters showed different responses to commonly used chemotherapeutic drugs. Cluster 1 exhibited lower IC50 values of nilotinib, paclitaxel and cisplatin and higher IC50 values of etoposide compared to cluster 2 (Fig. S1C). Mutation analysis indicated that cluster 2 had a higher tumor mutation burden than cluster 1 (Fig. 4A). Additionally, cluster 2 exhibited a higher number of various mutation types, including frame-shift deletion, frame-shift insertion, in-frame deletion, missense, nonsense, and silent mutations (Fig. 4B). Similarly, a relatively higher proportion of the samples in cluster 2 exhibited gene mutation, and the two genes with the highest mutation frequency were KRAS and TP53 in both clusters (Fig. 4C, D). Based on these results, cluster 1 exhibited higher immune cell abundance, higher immune checkpoint expression levels, and a lower mutation frequency than cluster 2.

### Functional annotation and molecular pathway analyses of the DEGs between the two clusters

Subsequently, we performed functional enrichment analysis, including GO and KEGG analyses and GSEA, and visualized the results with a dot plot (Fig. S2A, Fig. S2B) and GSEA plot (Fig. S2C, Fig. S2D). The main enriched biological functions and processes included the terms “immune response”, “immune cell differentiation and migration”, “cell activation”, “angiogenesis”, and “response to cytokine”, indicating a correlation with immune regulation and cancer development. In addition, the main enriched KEGG pathway terms of the DEGs were “chemokine signaling pathway”, “cell adhesion molecules”, and “JAK-STAT signaling pathway”; some immune differentiation-related pathways were also enriched. These results were consistent with the GO analysis results and indicated a potential role of the DEGs in pancreatic cancer.

### Establishment of a subtype-related risk model and prognostic nomogram

**Fig. 5:**
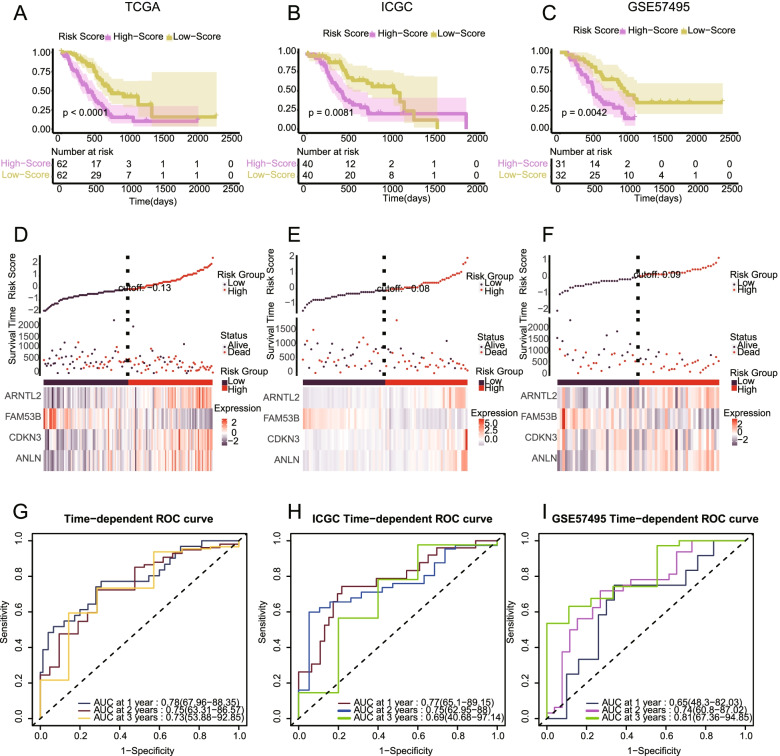
Establishment of a risk stratification model based on genes differentially expressed between the subtypes. **A-C** Patients with low risk scores exhibited longer survival times than those with high scores in the three cohorts (TCGA, ICGC, and GSE57495)**. D-F** Risk plots depicting the risk scores and survival times of each patients. A heatmap depicting the expression of core genes in pancreatic cancer patients is also shown (TCGA, ICGC, and GSE57495)**. G-I** The high AUC in the training set (TCGA) and validation set (ICGC and GSE57495) indicates the good predictive performance of our risk model

**Fig. 6:**
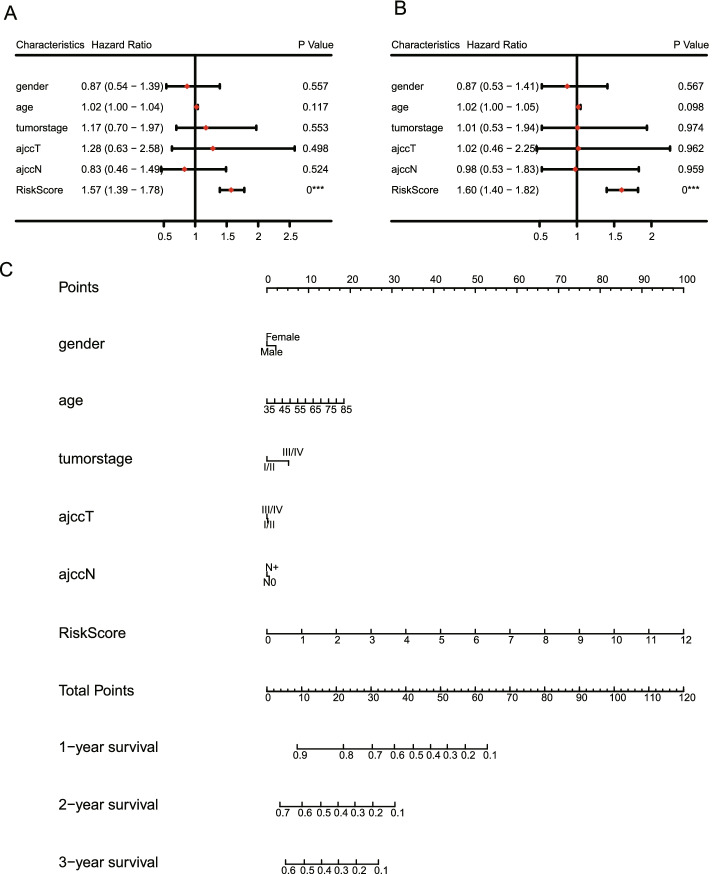
Clinical nomogram constructed based on the risk signature and clinical parameters. **A-B** Univariate and multivariate Cox regression analyses of our risk signature and clinical parameters (the asterisks represent p values; ***P < 0.001). **C** The clinical nomogram was constructed based on our risk signature and clinical parameters. The scales in the figure represent the single item scores corresponding to each variable under different values, and the total points indicate the total score after all variables have been assigned values

Given the remarkable differences in the characteristics of the RNA methylation-related subtypes, we further investigated the prognostic role of genes with differential expression between the subtypes. A total of 26 and 17 genes were identified after univariate Cox regression and LASSO regression combined with random forest analysis, respectively (Fig. S3A, Fig. S3B). In total, 4 differential genes were ultimately identified and utilized to construct a risk signature (Fig. S3B). The model formula is as follows: risk score = 1.0746*exp. (CDKN3)-0.9659*exp. (FAM53B)-0.5651*exp.(ANLN) + 0.7498*exp.(ARNTL2). The correlation heatmap shows a high correlation between the screened genes (Fig. S3C). The prognostic utility of the risk signature was determined in a TCGA training cohort and validated in external datasets: GSE57495 and ICGC. The results indicated that patients with low risk scores exhibited longer survival times in all cohorts, with *p* values of 0.001, 0.0081, and 0.0042 in the TCGA, GSE57495, and ICGC cohorts, respectively (Fig. 5A, B, C). The risk plots depict the risk score and survival time of each patient (Fig. 5D, E, F). ROC curve analysis was used to test the reliability and stability of the risk signature (Fig. 5G, H, I). The high AUC values in the training set and validation set indicated the good predictive performance of our model (generally greater than 0.7). Furthermore, we compared the prognostic value of our risk signature with those of some important clinical parameters. Univariate and multivariate Cox regression analyses indicated that our risk score was an independent prognostic indicator in pancreatic cancer (*p* value< 0.001) (Fig. 6A, B). A clinical nomogram was constructed based on our risk signature and included the clinical parameters age, sex, AJCC T stage, AJCC N stage, and tumor stage (Fig. 6C). Calibration curve analysis and decision curve analysis (DCA) verified the predictive utility of our nomogram (Fig. S4A, Fig. S4B).

### Ability of the RNA methylation-based model to reflect the immune landscape and drug sensitivity

**Fig. 7:**
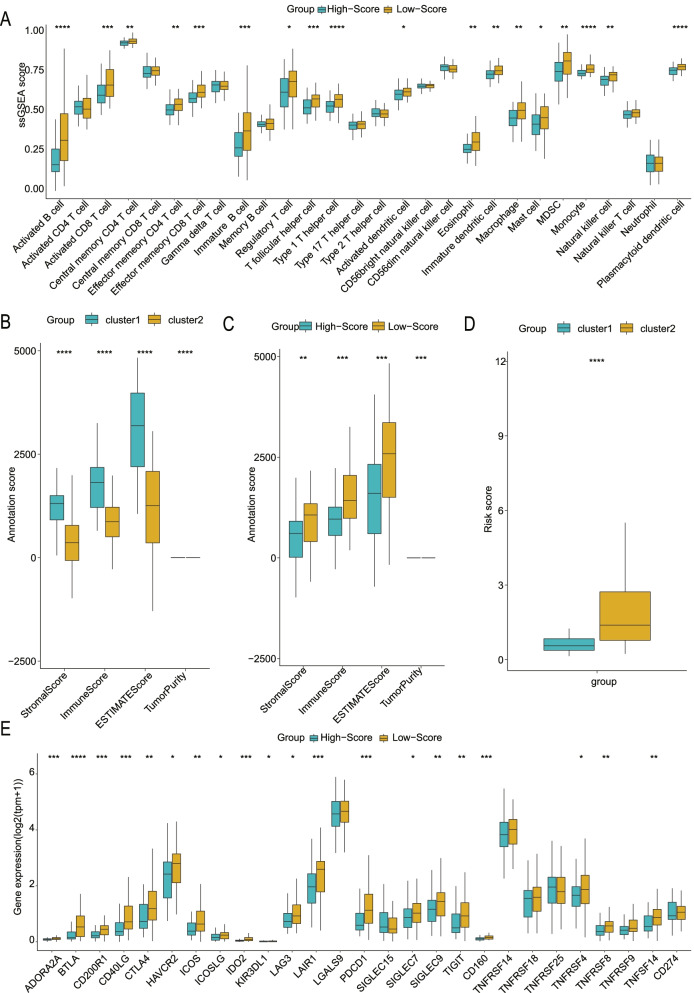
Analysis of the ability of the RNA methylation-related signature to reflect the immune landscape. **A** The low-score group had higher immune cell abundance than the high-score group for all immune cells (all P < 0.05). Comparisons were made with the Wilcoxon test, the lines in the boxes represent medians, and the asterisks represent p values. *P < 0.05, **P < 0.01, ***P < 0.001, ****P < 0.0001, ns P > 0.05. **B-D** The high-score group exhibited a lower stromal score, estimate score, and immune score and higher tumor purity than the low-score group, consistent with the features of cluster 2. Comparisons were made with the Wilcoxon test, the lines in the boxes represent medians, and the asterisks represent p values. *P < 0.05, **P < 0.01, ***P < 0.001, ****P < 0.0001, ns P > 0.05. **E** The low-score group exhibited higher expression levels of immune checkpoints than the high-score group. Comparisons were made with the Wilcoxon test, the lines in the boxes represent medians, and the asterisks represent p values. *P < 0.05, **P < 0.01, ***P < 0.001, ****P < 0.0001, ns P > 0.05

To further explore the practical application of our subtype-related risk model in pancreatic cancer, we investigated its ability to reflect immune infiltration and chemotherapeutic sensitivity. ssGSEA analysis indicated that the low-score group had higher immune cell abundance than the high-score group (Fig. 7A). The level of M0 macrophages was higher in the high-score group than in the low-score group (CIBERSORT); macrophages can be polarized into M1 or M2 macrophages, which play opposite roles (Fig. S5A) [75]. In addition, the high-score group exhibited a lower stromal score, estimate score, and immune score and a higher tumor purity, which was consistent with the features of cluster 2 (Fig. 7B, C). Interestingly, cluster 2 exhibited a higher risk score than cluster 1, suggesting that cluster 2 may have a higher risk and worse prognosis (Fig. 7D). Additionally, the low-score group exhibited high expression levels of all immune checkpoints, indicating increased potential for immunotherapy in this group (Fig. 7E). We estimated the chemotherapy sensitivity of the different risk groups. The low-score group had a higher IC50 value of etoposide but a lower IC50 value of cisplatin (Fig. S5B).

### The risk model had better predictive ability and accuracy than other published models

**Fig. 8:**
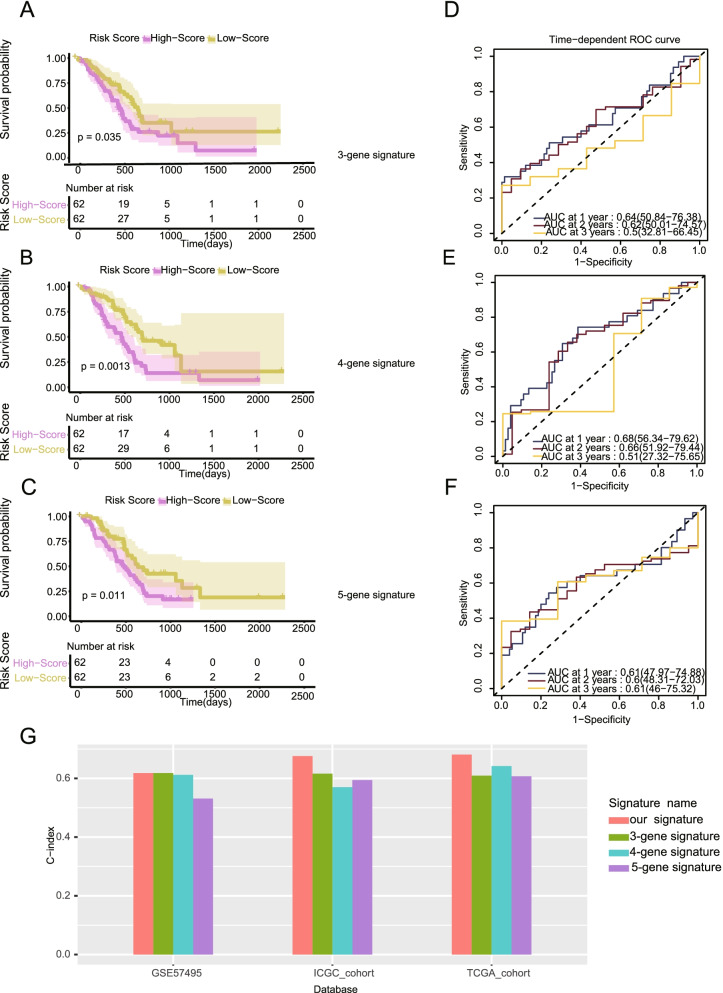
The risk model exhibited better predictive performance and accuracy than other published models (TCGA). **A-C** The model exhibited a better ability to predict survival than published signatures. **D-F**. The model exhibited a higher AUC than published signatures. **G** The model exhibited a higher c-index than published models in for all cohorts

To further prove the superiority of our risk model, we compared our model with some published methylation-related models, including a 3-gene signature, a 4-gene signature, and a 5-gene signature. Survival curve, ROC curve, and c-index analyses were used to evaluate the models. The analyses were based on TCGA data, and the result indicated that our model exhibited a better ability to predict survival and to distinguish the high- and low-risk groups than other models (Fig. 8A, B, C). The ROC curve analysis suggested that our model had higher stability and accuracy (Fig. 8D, E, F). We also performed these analyses in the ICGC and GSE57495 cohorts, and the results validated our conclusion (Fig. S6, Fig. S7). In addition, we comprehensively compared the c-indexes of various models in the TCGA, ICGC, and GSE57495 cohorts. Our model exhibited a higher c-index than other published models for all cohorts (Fig. 8G).

### Validation of the accuracy of our risk stratification model with cytology and histology experiments

We cultured a normal pancreatic ductal epithelial cell line (HPDE) and four pancreatic cancer cell lines (SW 1990, BxPC-3, CFPAC-1, and PANC-1), extracted RNA from the cells, and performed qRT-PCR to determine the expression profiles of key prognostic genes (ANLN, ARNTL2, CDKN3, and FAM53B) and RNA methylation regulators. The results (Fig. S8) indicated that the mRNA levels of ANLN, ARNTL2, CDKN3, and FAM53B in the cancer cell lines (SW 1990, BxPC-3, CFPAC-1, and PANC-1) were significantly higher than those in the normal cell line (HPDE), indicating their prognostic value. In addition, the expression levels of ANLN, ARNTL2, CDKN3, and FAM53B were upregulated in cancer tissues from our cancer center (Fig. S9A). The expression levels of most RNA methylation regulators were also elevated in the tumor cell lines, suggesting that pancreatic cancer tumor cells may have higher levels of RNA methylation (Fig. S10). Our model also performed well in a FUSCC cohort. The survival time of high-risk patients was significantly lower than that of low-risk patients (*P* value < 0.05), with AUCs of 0.66 and 0.75 for one and two years, respectively (Fig. S9B, Fig. S9C).

### CDKN3 significantly promotes the proliferation and migration of pancreatic cancer cells

**Fig. 9:**
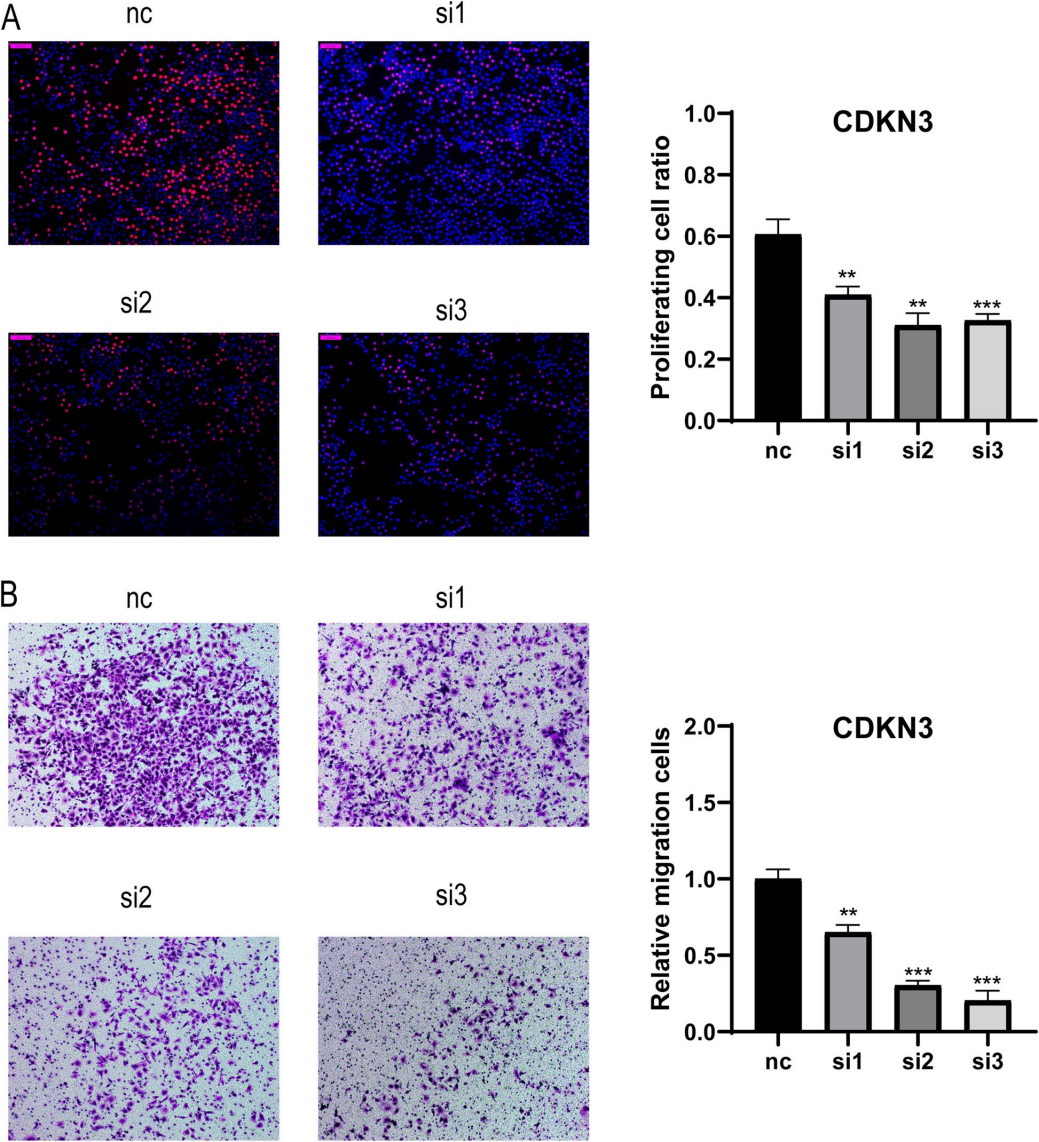
CDKN3 significantly promotes the proliferation and migration of pancreatic cancer cells. **A** CDKN3 silencing reduced the proliferation of PANC-1 cells. **B** Knockdown of CDKN3 inhibited the migration of PANC-1 cells. Comparisons were made with the unpaired t test, and the asterisks represent p values. *P < 0.05, **P < 0.01, ***P < 0.001

CDKN3 expression was silenced in PANC-1 cells with small-interfering RNA. The apoptosis rate of pancreatic cancer cells was not remarkably influenced by gene silencing (Fig. S11). The proliferation of CDKN3-silenced cells was decreased compared to that of control cells (Fig. 9A). Additionally, CDKN3-silenced cells exhibited lower migration ability in Transwell migration experiments (Fig. 9B).

## Discussion

Recently, epigenetic alterations triggered by RNA methylation have become a research focus, and such alterations may be associated with the initiation and progression of malignances. In the field of pancreatic cancer RNA methylation research, some scientists have illuminated the potential role of the methylation modifying enzyme METTL3 in modulating proliferation, invasion and therapeutic sensitivity [76], and efforts to investigate the internal mechanism have been made. Some studies have also explored the prognostic role of RNA methylation in pancreatic cancer from the perspective of m6A and constructed risk classification models [74]. However, no risk stratification or prognostic models have been generated based on holistic bioinformatics exploration of RNA methylation. In this research, we identified methylation-related subtypes of pancreatic cancer based on the expression patterns of 38 RNA methylation regulators and explored the immune, mutation, and chemosensitivity characteristics of the different subtypes. Additionally, a risk stratification signature based on DEGs was established to further explore differences in prognosis and immunotherapy and chemotherapy efficacy between the two risk groups.

Aberrant RNA methylation plays an essential role in the occurrence and progression of pancreatic cancer [74, 77]. Our results suggest that stratifying pancreatic cancer patients based on RNA methylation profiles may be useful for improving patient outcomes. Two subtypes with remarkable survival differences were identified by consensus clustering, indicating the prognostic role of RNA methylation in pancreatic cancer. In addition, we shed light on the distinct immune, chemotherapy sensitivity, and mutation characteristics of the two clusters. Cluster 1 exhibited a significantly higher abundance of almost all immune cells than cluster 2, suggesting that cluster 1 features robust immune infiltration. Similarly, cluster 1 had higher expression of the majority of immune checkpoints than cluster 2, revealing that cluster 1 is more likely to benefit from immunotherapy. Additionally, chemotherapeutic sensitivity analysis indicated that there were significant differences in the sensitivity of four drugs between the different subgroups, suggesting that our RNA methylation-based stratification method has potential for guiding chemotherapy drug selection. Further mutation investigation revealed a higher mutation burden and higher number of mutations in cluster 2, which was consistent with the survival analysis. Overall, we identified 2 subtypes with considerable differences and verified the poor biological behaviors and prognosis of cluster 2, providing guidance for predicting clinical outcomes and drug selection. Further experiments are needed to explore the clinical value of the clustering and validate its performance.

We established an accurate and feasible risk stratification method based on DEGs. The use of multiple screening methods, including univariate Cox regression, LASSO regression, random forest, and multivariate Cox regression analyses, ensures the robustness of our results. A four-gene risk signature was ultimately identified and exhibited an optimal ability to predict survival. Univariate and multivariate Cox analyses indicated that our risk score was an independent prognostic indicator. Additionally, comparison of our risk model with other published risk signatures confirmed the superiority of our model. These findings suggest that our risk stratification method based on RNA methylation is useful.

Studies of the immune microenvironment have increased with the rise of immunotherapy and targeted therapy. Pancreatic cancer exhibits an immunosuppressive microenvironment that includes various immunosuppressive cells (tumor-associated macrophages, myeloid-derived suppressor cells (MDSCs), and regulatory T cells), which may contribute to the failure of immune therapy [78]. Surprisingly, RNA methylation may be involved in the modulation of immune cells and immune microenvironments [79]. Some scientists have demonstrated that elevated expression of METTL3 promotes the proliferation of CD33+ MDSCs, leading to the progression of cervical cancer [80]. In our study, comprehensive analysis of different risk groups and clusters was performed, and immune infiltration levels were analyzed. The low-risk group exhibited higher immune cell abundance and immune checkpoint levels than the high-risk group, indicating that the low-risk group is more likely to benefit from therapy and have a good prognosis. Additionally, the characteristics of the low-risk group were consistent with those of cluster 1: higher stromal score, estimate score, and immune score and lower tumor purity. In conclusion, the risk stratification model is more accurate and practical for subgroup classification and has a robust ability to predict immunotherapy efficacy and prognosis. Subsequent experiments are needed to validate its utility in clinical practice.

Our study has some strengths and limitations. First, our model was tested in multiple datasets and is thus reliable. Second, we verified the expression differences and prognostic utility of the hub genes in our own cohort. A limitation of our research is potential differences in sample standardization methods between various datasets. Further verification with data from more centers and larger sample sizes is needed.

## Conclusion

In conclusion, we systematically analyzed the expression patterns of RNA methylation regulators in pancreatic cancer and identified two molecular subtypes with completely different characteristics. We constructed a risk stratification model based on these subtypes that performed better than published risk models. Our study is the first to include comprehensive bioinformatics and prognostic analysis of RNA methylation in pancreatic cancer, and we hope the results will provide references for clinical practice.

## Supplementary Information

**Additional file 1.**

**Additional file 2.**

**Additional file 3.**

**Additional file 4.**

**Additional file5.**

**Additional file 6.**

**Additional file 7.**

**Additional file 8.**

**Additional file 9.**

**Additional file 10.**

**Additional file 11.**

**Additional file 12.**

**Additional file 13.**

## Data Availability

All datasets used in the present study are publicly available: GEO: https://www.ncbi.nlm.nih.gov/geo/; TCGA: https://portal.gdc.cancer.gov/); ICGC: https://icgcportal.genomics.cn/.
