# Supplementary figures and images for: Comprehensive analysis of the prognosis and immune infiltration landscape of RNA methylation-related subtypes in pancreatic cancer
Source: BMC Cancer. 2022 Jul 21;22:804. doi: 10.1186/s12885-022-09863-z (PMC9306066; doi:10.1186/s12885-022-09863-z)

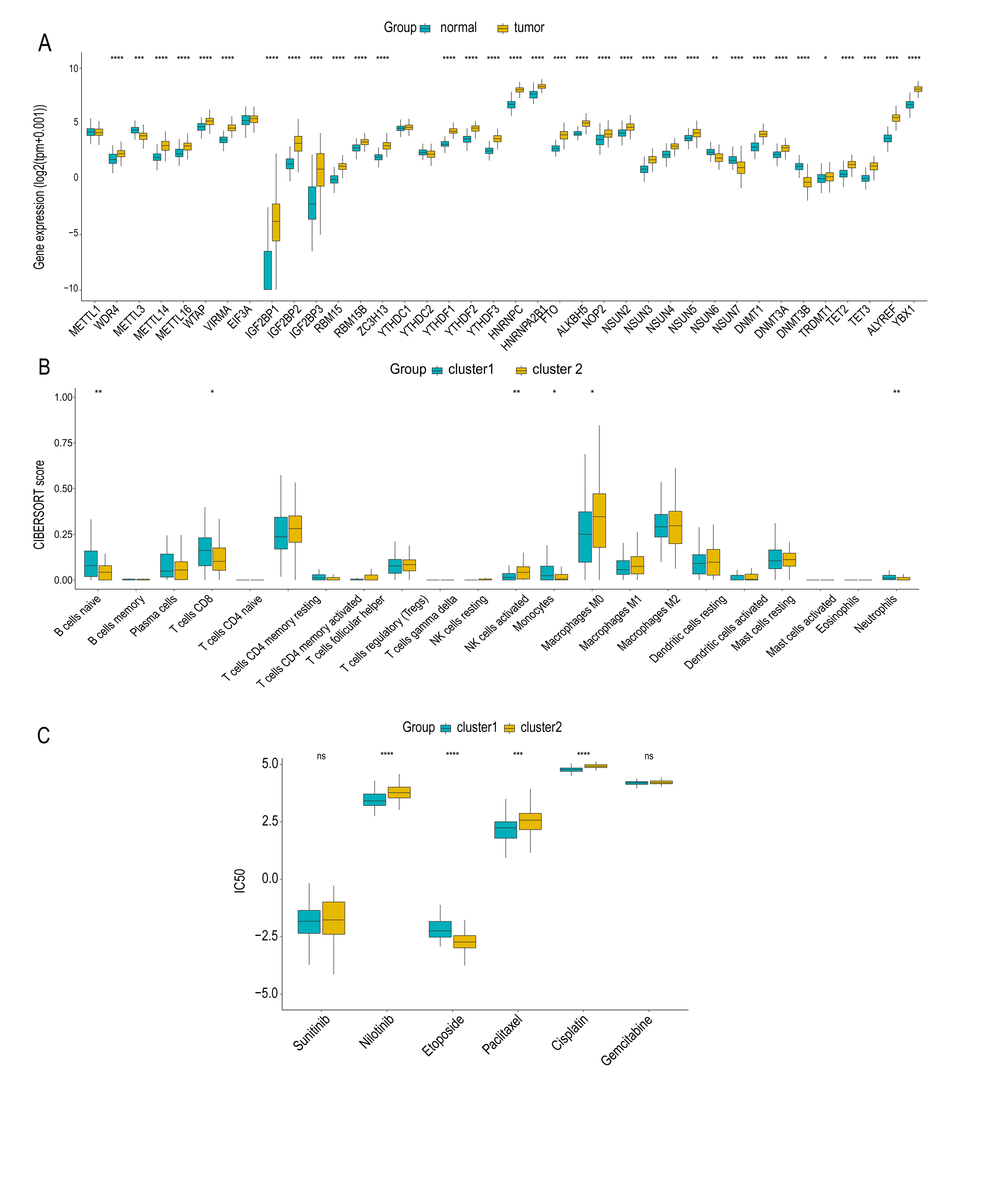

Supplement: Supplementary file 1 — Additional file 1. [file 12885_2022_9863_MOESM1_ESM.tif]

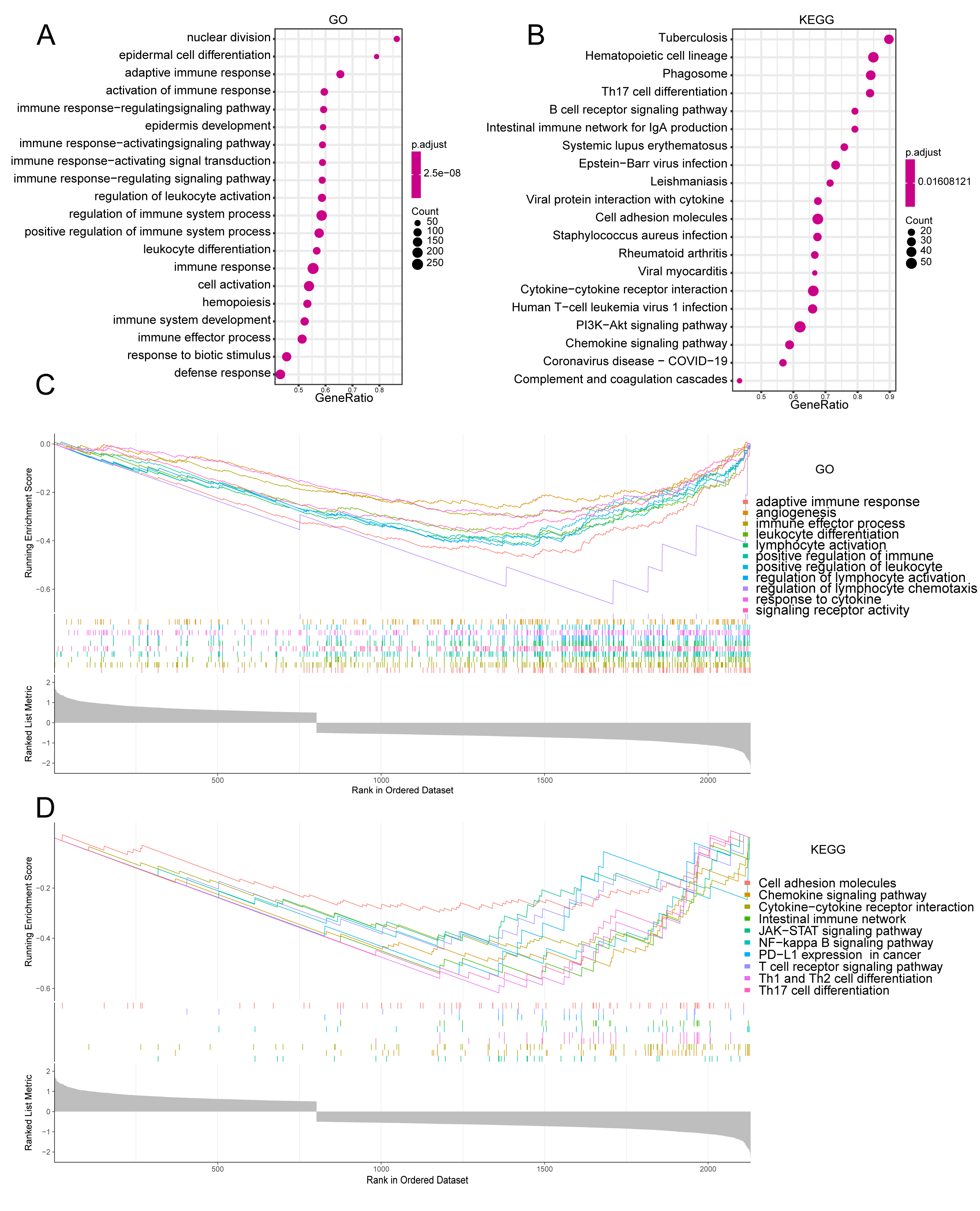

Supplement: Supplementary file 2 — Additional file 2. [file 12885_2022_9863_MOESM2_ESM.tif]

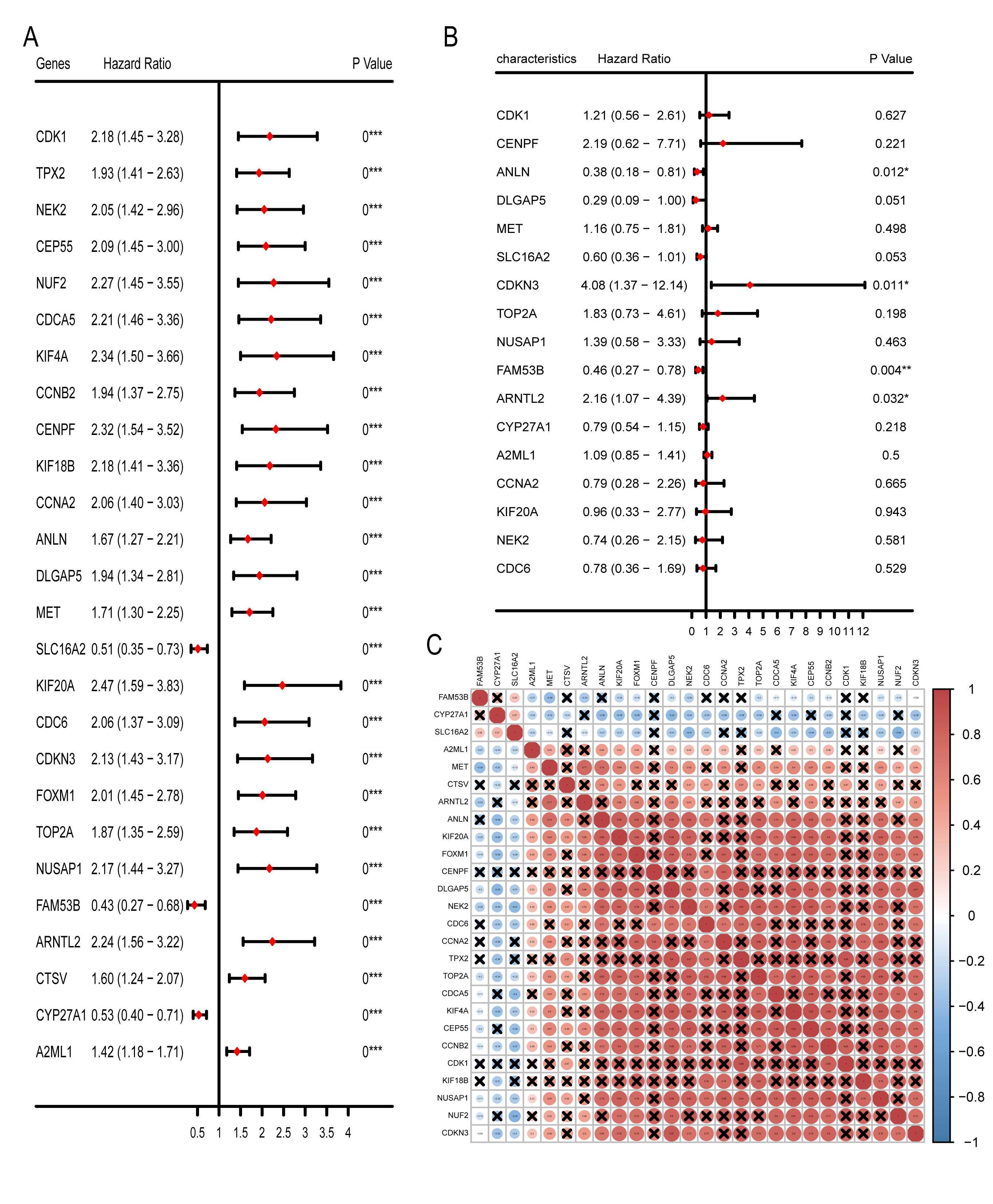

Supplement: Supplementary file 3 — Additional file 3. [file 12885_2022_9863_MOESM3_ESM.tif]

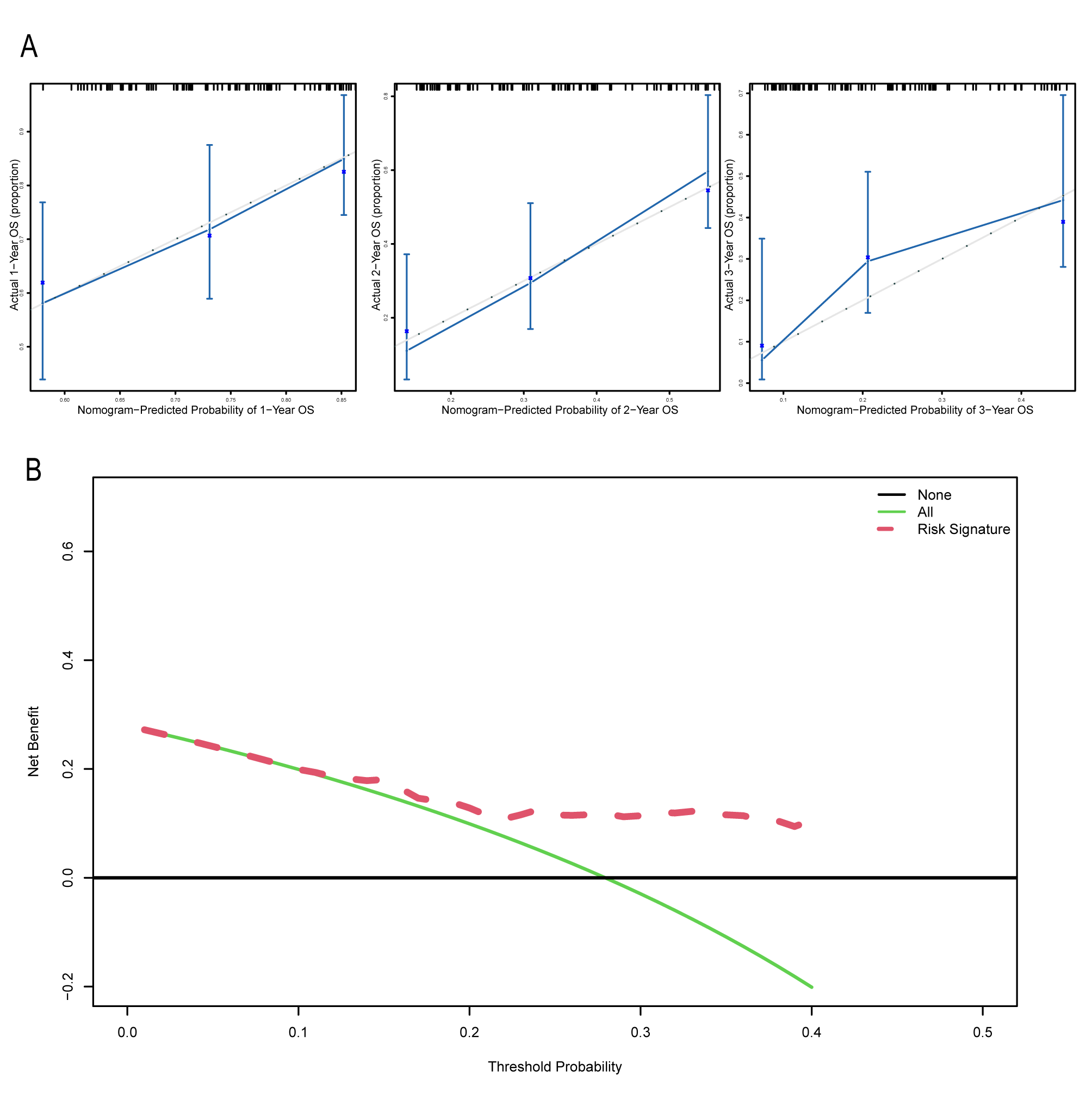

Supplement: Supplementary file 4 — Additional file 4. [file 12885_2022_9863_MOESM4_ESM.tif]

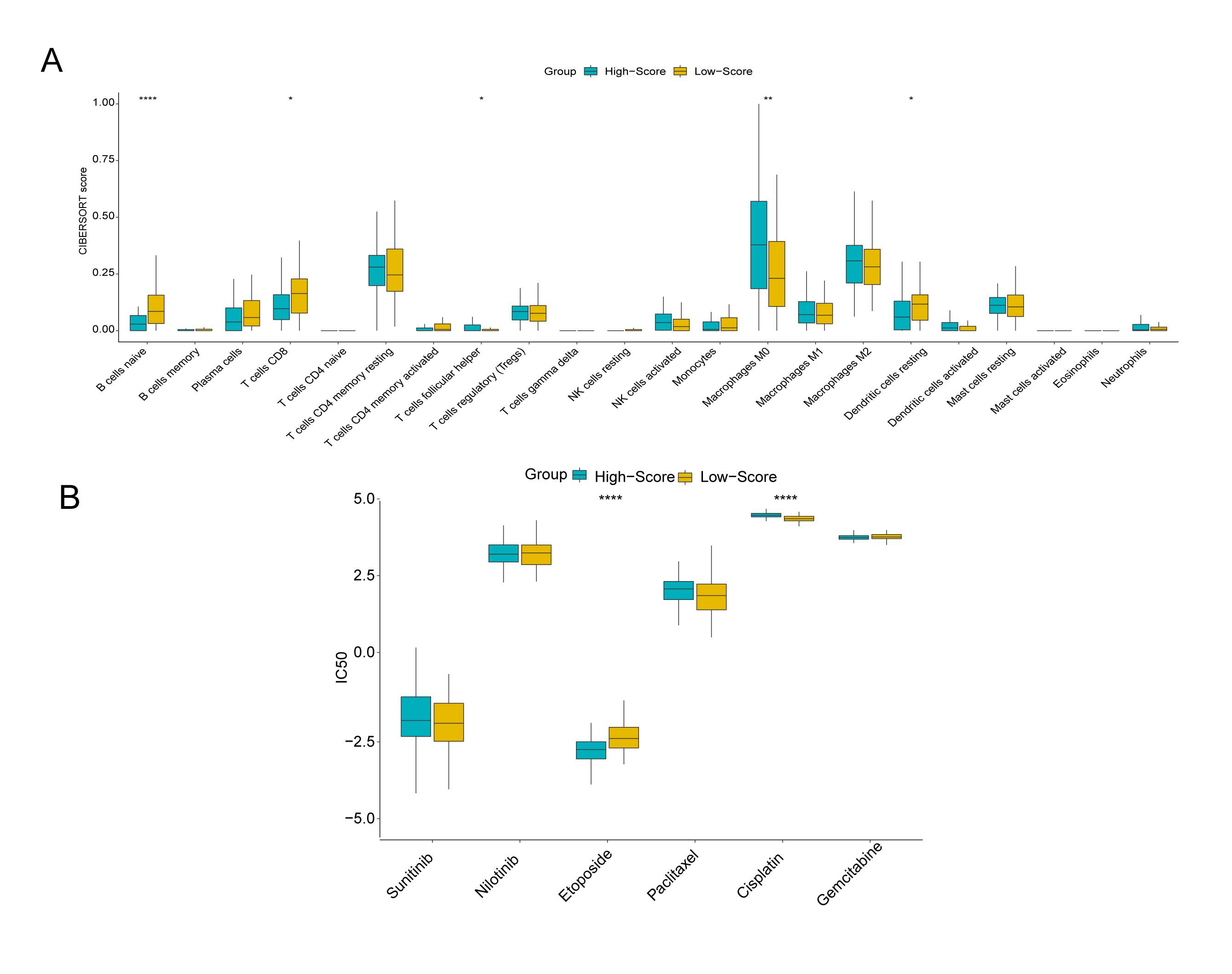

Supplement: Supplementary file 5 — Additional file5. [file 12885_2022_9863_MOESM5_ESM.tif]

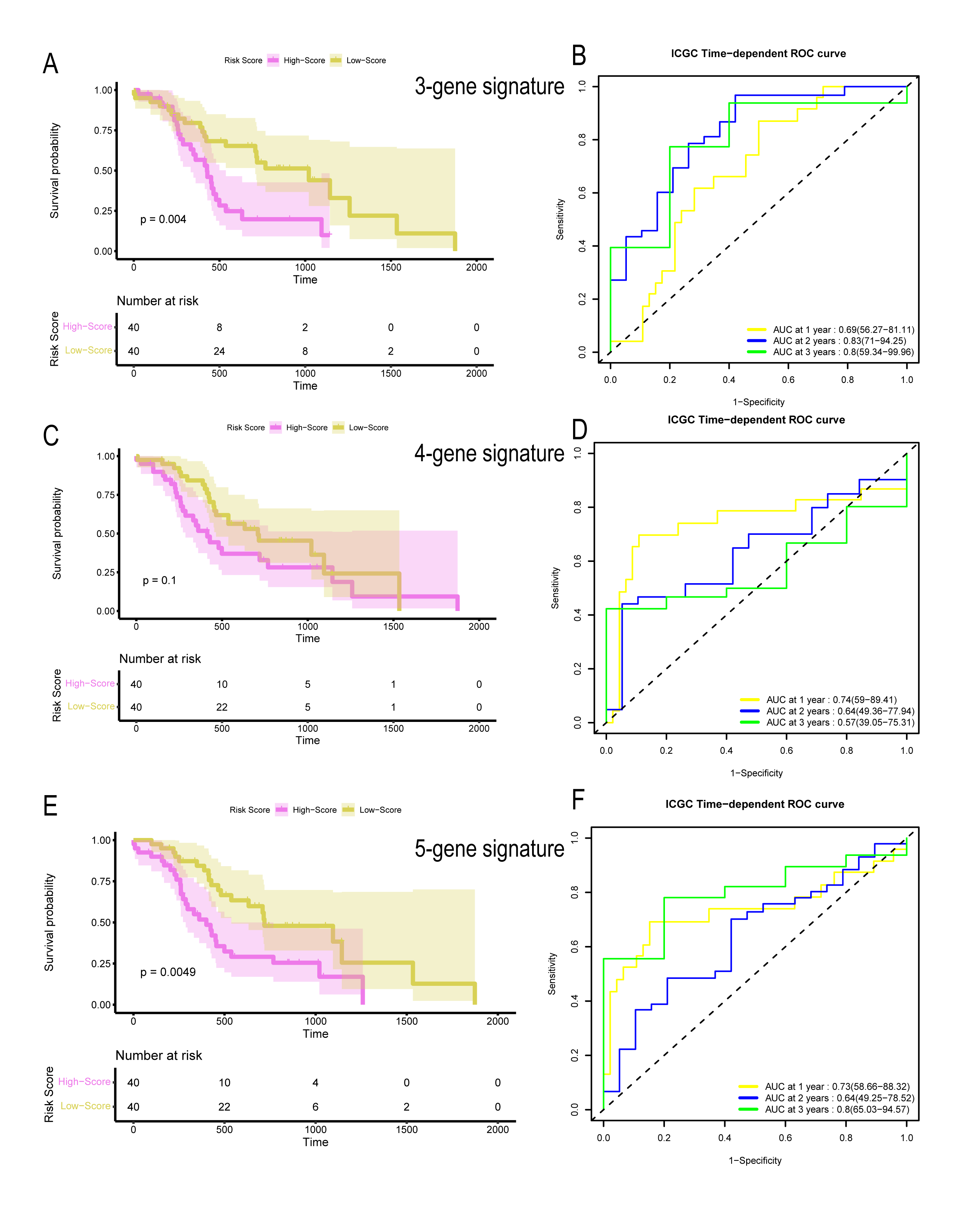

Supplement: Supplementary file 6 — Additional file 6. [file 12885_2022_9863_MOESM6_ESM.tif]

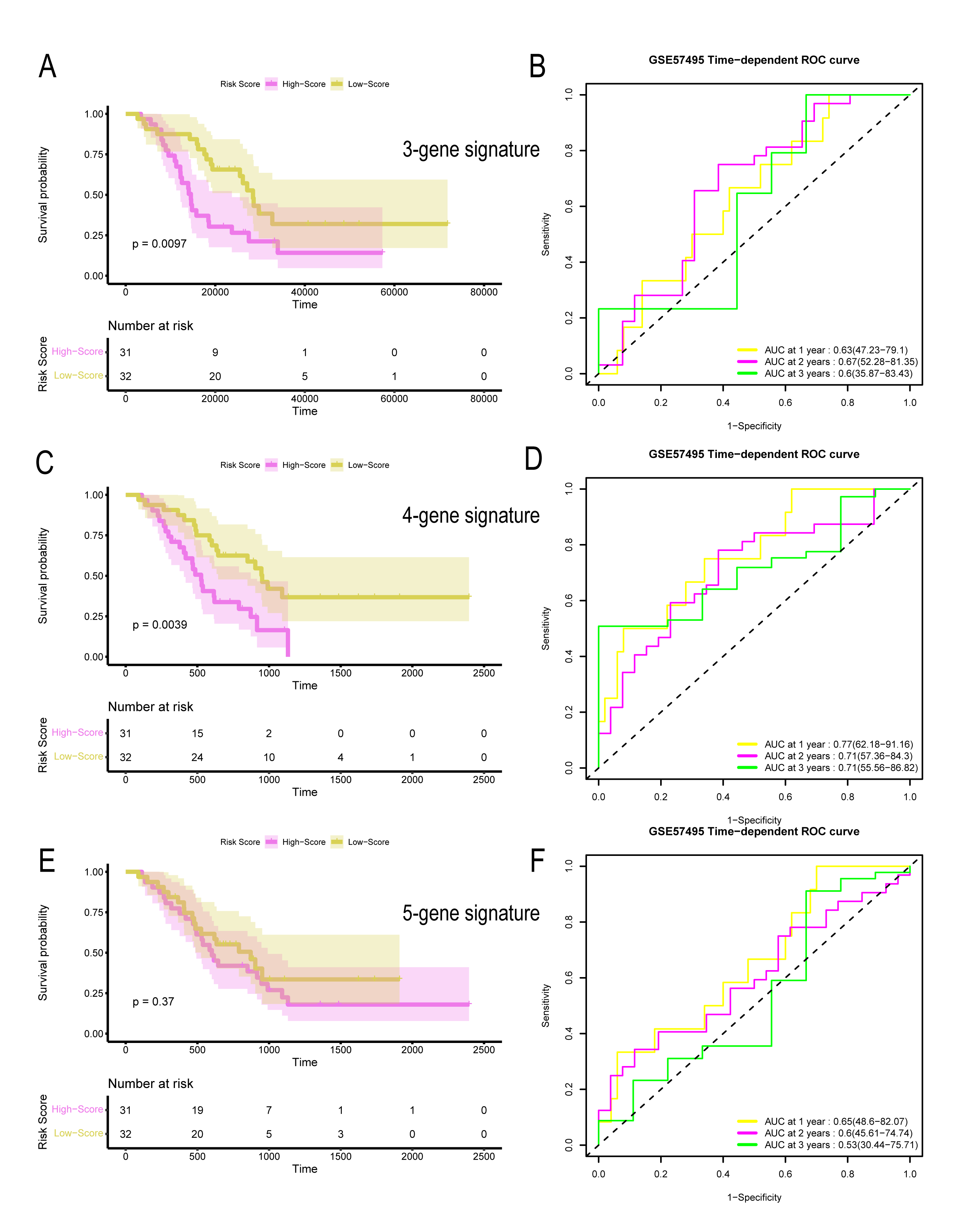

Supplement: Supplementary file 7 — Additional file 7. [file 12885_2022_9863_MOESM7_ESM.tif]

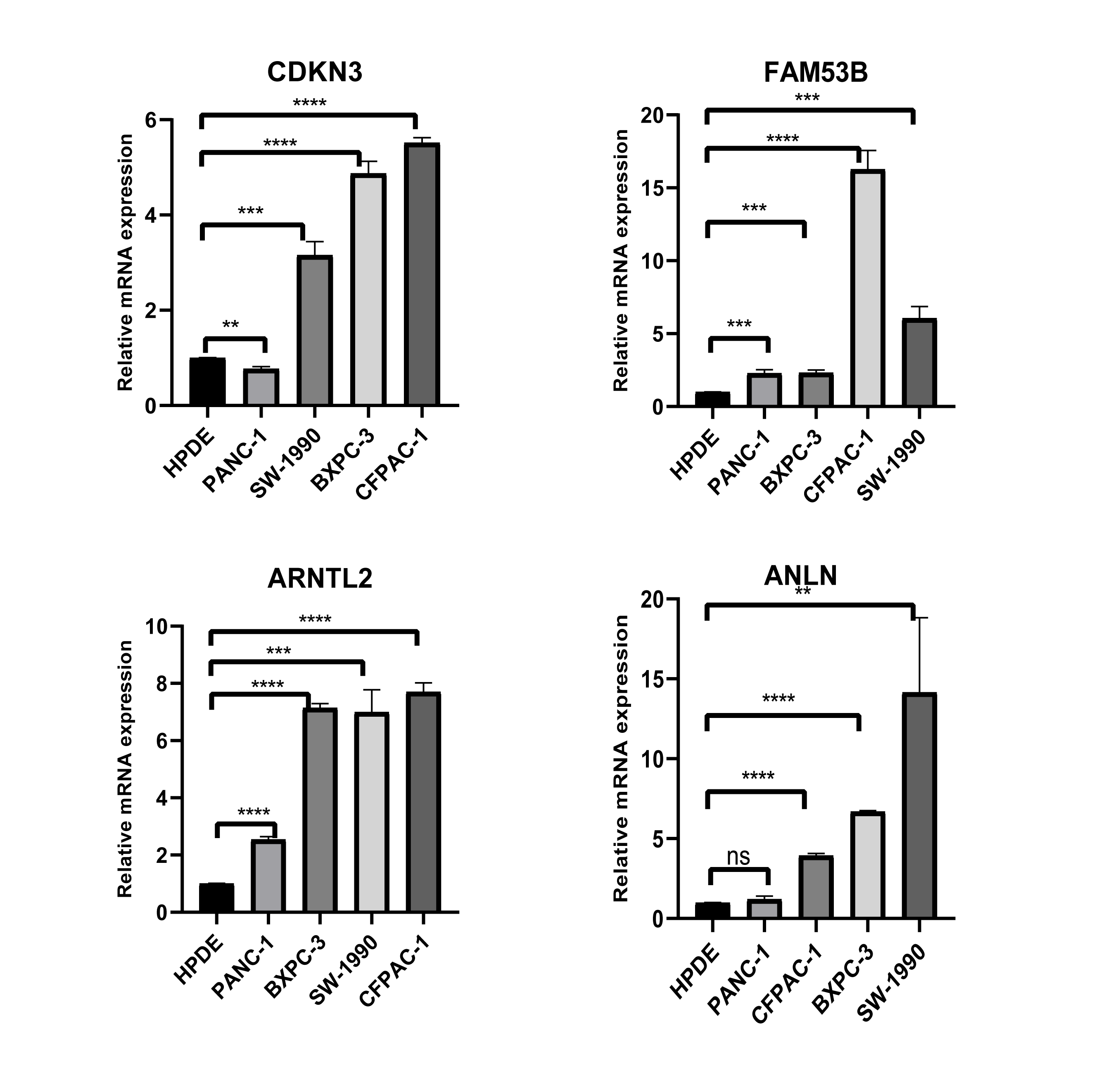

Supplement: Supplementary file 8 — Additional file 8. [file 12885_2022_9863_MOESM8_ESM.tif]

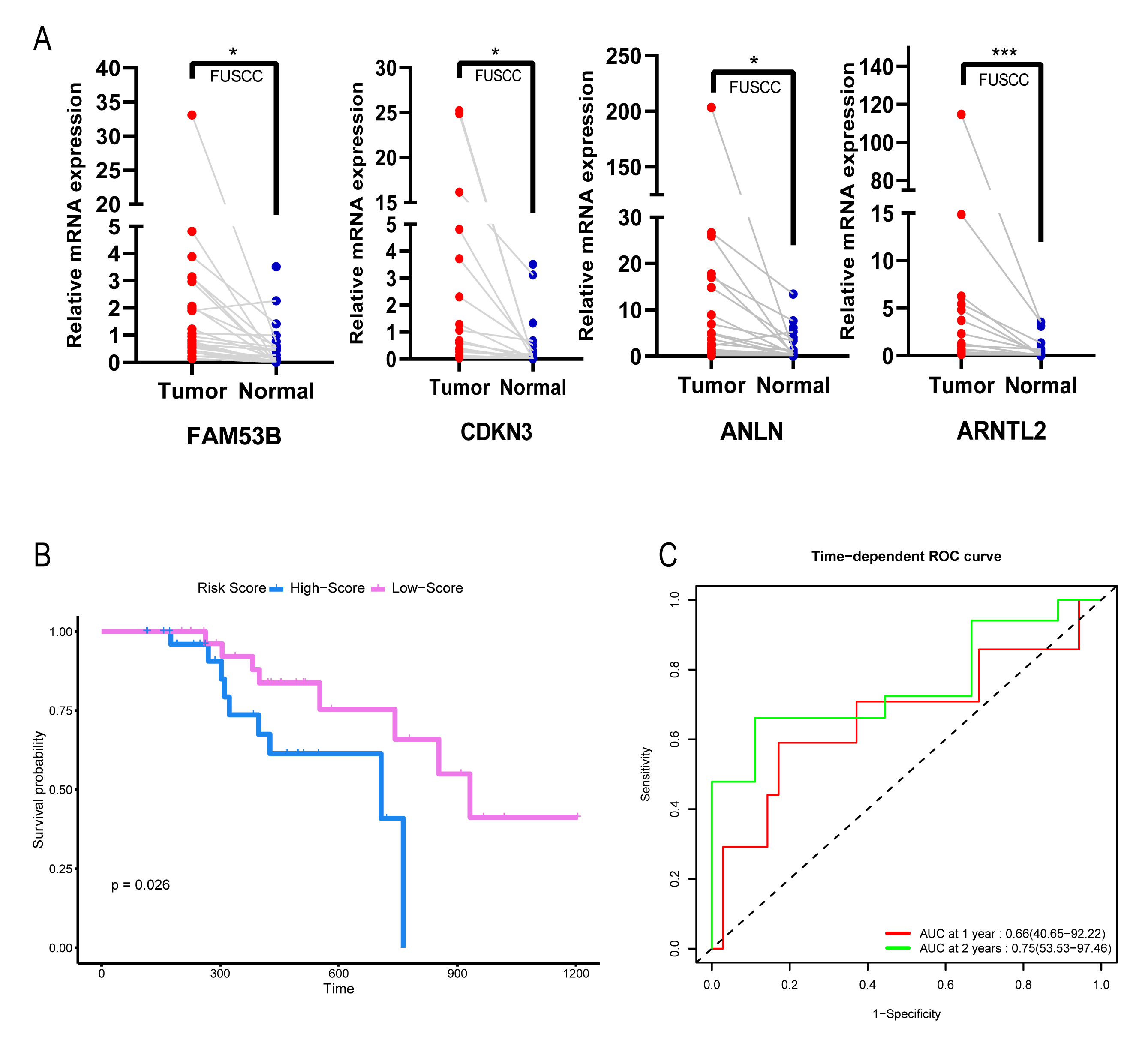

Supplement: Supplementary file 9 — Additional file 9. [file 12885_2022_9863_MOESM9_ESM.tif]

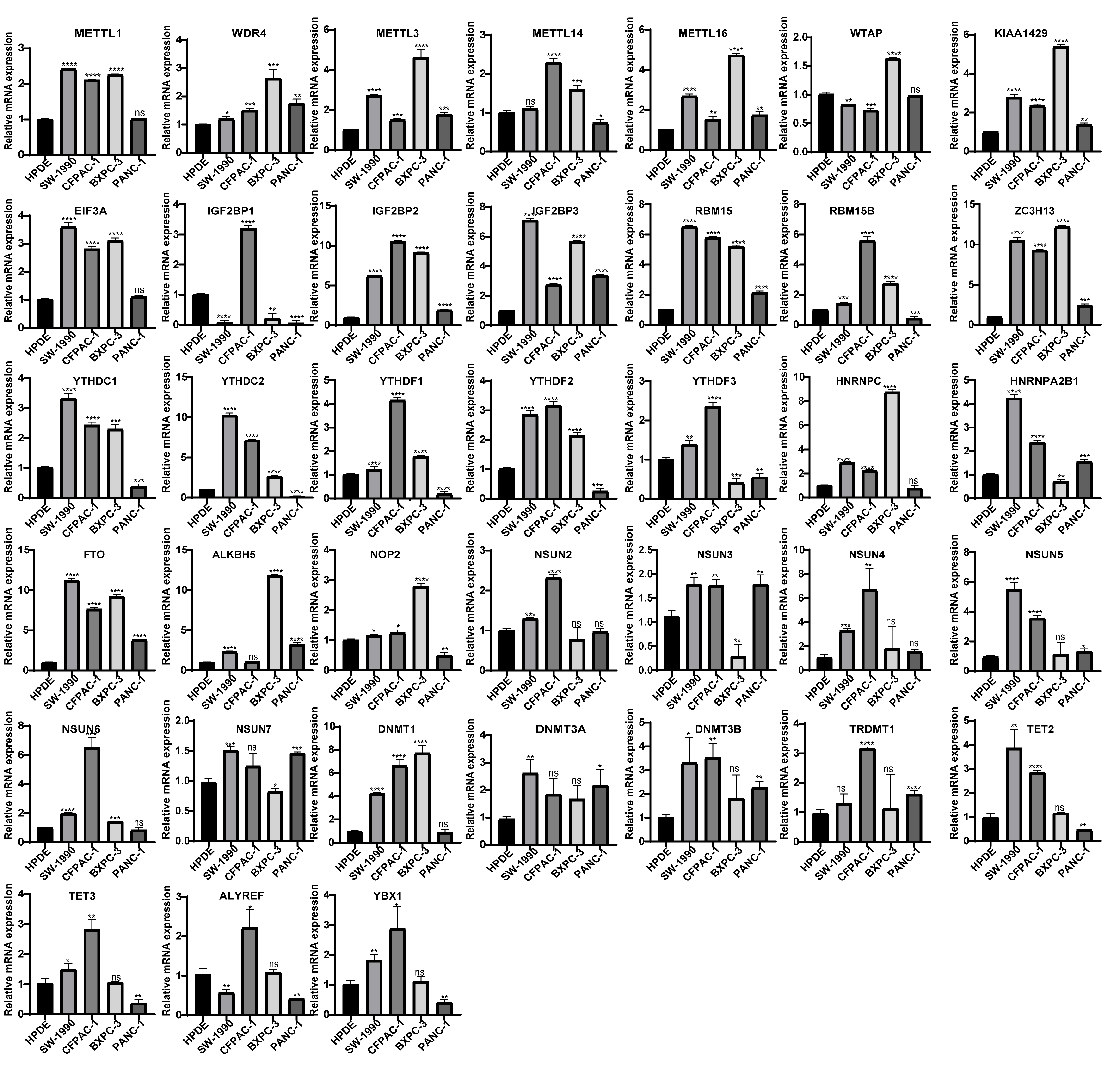

Supplement: Supplementary file 10 — Additional file 10. [file 12885_2022_9863_MOESM10_ESM.tif]

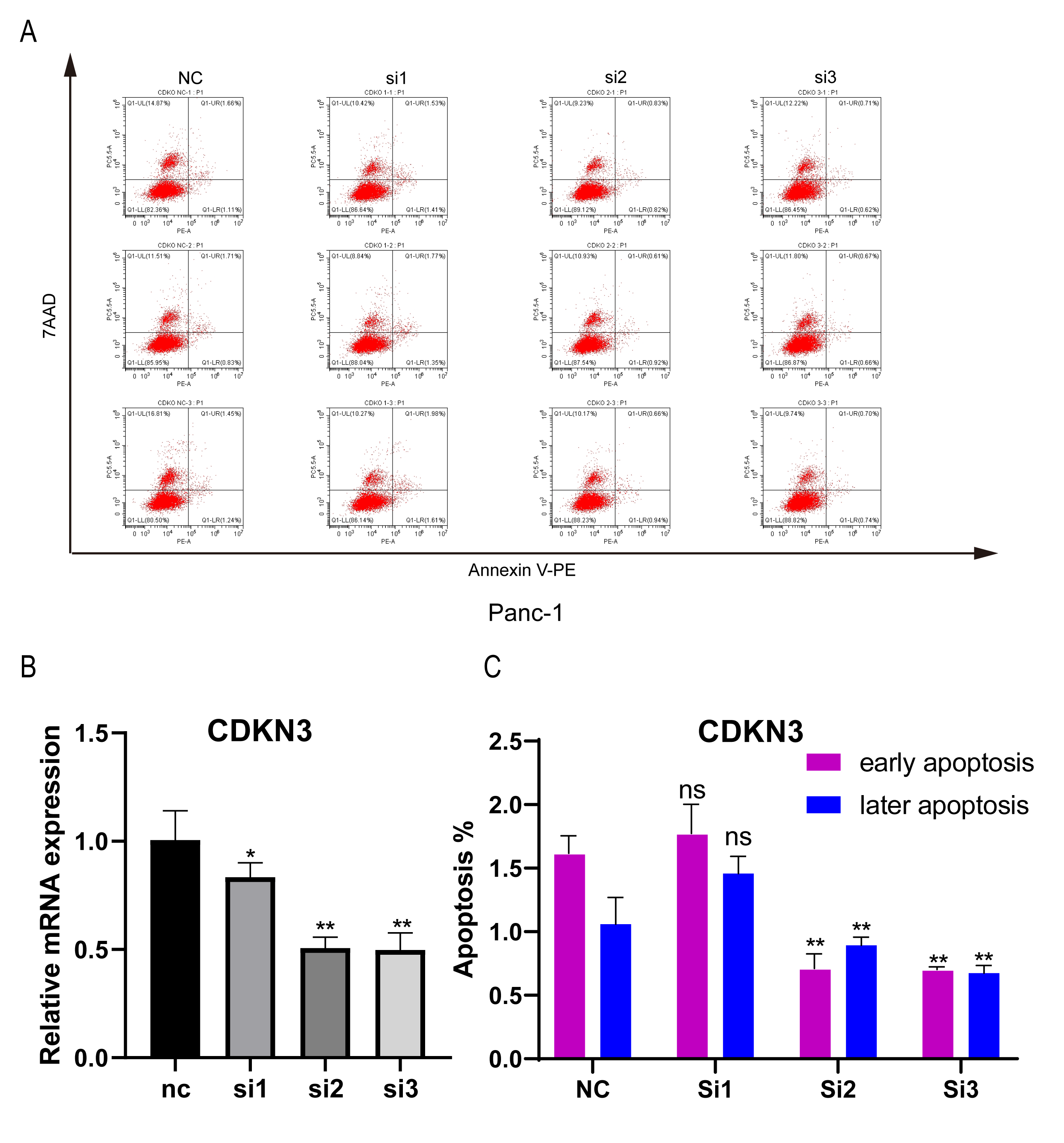

Supplement: Supplementary file 11 — Additional file 11. [file 12885_2022_9863_MOESM11_ESM.tif]
