# Supplementary material for: Comprehensive analysis of the prognosis and immune infiltration landscape of RNA methylation-related subtypes in pancreatic cancer
Source: BMC Cancer. 2022 Jul 21;22:804. doi: 10.1186/s12885-022-09863-z (PMC9306066; doi:10.1186/s12885-022-09863-z)
Supplement: Supplementary file 12 — Additional file 12. [file 12885_2022_9863_MOESM12_ESM.docx]

Supplementary table 1. Immune checkpoint in the present study.

| Immune checkpoints | | |  |  |
| --- | --- | --- | --- | --- |
| ADORA2A |  |  |  |  |
| BTLA |  |  |  |  |
| CD200R1 |  |  |  |  |
| CD40LG |  |  |  |  |
| CTLA4 |  |  |  |  |
| HAVCR2 |  |  |  |  |
| ICOS |  |  |  |  |
| ICOSLG |  |  |  |  |
| IDO2 |  |  |  |  |
| KIR3DL1 |  |  |  |  |
| LAG3 |  |  |  |  |
| LAIR1 |  |  |  |  |
| LGALS9 |  |  |  |  |
| PDCD1 |  |  |  |  |
| SIGLEC15 |  |  |  |  |
| SIGLEC7 |  |  |  |  |
| SIGLEC9 |  |  |  |  |
| TIGIT |  |  |  |  |
| CD160 |  |  |  |  |
| TNFRSF14 |  |  |  |  |
| TNFRSF18 |  |  |  |  |
| TNFRSF25 |  |  |  |  |
| TNFRSF4 |  |  |  |  |
| TNFRSF8 |  |  |  |  |
| TNFRSF9 |  |  |  |  |
| TNFSF14 |  |  |  |  |
| CD274 |  |  |  |  |
