# Supplementary material for: Comprehensive analysis of the prognosis and immune infiltration landscape of RNA methylation-related subtypes in pancreatic cancer
Source: BMC Cancer. 2022 Jul 21;22:804. doi: 10.1186/s12885-022-09863-z (PMC9306066; doi:10.1186/s12885-022-09863-z)
Supplement: Supplementary file 13 — Additional file 13. [file 12885_2022_9863_MOESM13_ESM.docx]

Supplementary table 2. Primers used in the present study.

| ID | Primary | Reverse |
| --- | --- | --- |
| ANLN | TGCCAGGCGAGAGAATCTTC | CGCTTAGCATGAGTCATAGACCT |
| ARNTL2 | TGGCGCGTAAACTGGACAAA | CCTCTTTCACATCCAACCACAAA |
| CDKN3 | TCCGGGGCAATACAGACCAT | GCAGCTAATTTGTCCCGAAACTC |
| FAM53B | GAGCTGACTCCATTGCATGTG | CAGGTCTCGCCATCTGTCAT |
| GAPDH | CAGGAGGCATTGCTGATGAT | GAAGGCTGGGGCTCATTT |
| METTL1 | GGCAACGTGCTCACTCCAA | CACAGCCTATGTCTGCAAACT |
| WDR4 | CCACCTCCATAGCAAGCAGTG | ACGCTTACTGTCATCGGTTAAAG |
| METTL3 | TTGTCTCCAACCTTCCGTAGT | CCAGATCAGAGAGGTGGTGTAG |
| METTL14 | AGTGCCGACAGCATTGGTG | GGAGCAGAGGTATCATAGGAAGC |
| METTL16 | CTCTGACGTGTACTCTCCTAAGG | TACCAGCCATTCAAGGTTGCT |
| WTAP | CTTCCCAAGAAGGTTCGATTGA | TCAGACTCTCTTAGGCCAGTTAC |
| KIAA1429 | AAGTGCCCCTGTTTTCGATAG | ACCAGACCATCAGTATTCACCT |
| EIF3A | GCCGGAAAATGCCCTCAAAC | TGGTTCGTGTATCTTTTGCCAT |
| IGF2BP1 | GCGGCCAGTTCTTGGTCAA | TTGGGCACCGAATGTTCAATC |
| IGF2BP2 | AGTGGAATTGCATGGGAAAATCA | CAACGGCGGTTTCTGTGTC |
| IGF2BP3 | TATATCGGAAACCTCAGCGAGA | GGACCGAGTGCTCAACTTCT |
| RBM15 | ACGACCCGCAACAATGAAG | GGAAGTCGAGTCCTCACCAC |
| ZC3H13 | TCTGATAGCACATCCCGAAGA | CAGCCAGTTACGGCACTGT |
| YTHDC1 | AACTGGTTTCTAAGCCACTGAGC | GGAGGCACTACTTGATAGACGA |
| YTHDC2 | AATTTGGGACGCATCATAAAGCC | CTCTGGTCCCCGTATCGGA |
| YTHDF1 | ACCTGTCCAGCTATTACCCG | TGGTGAGGTATGGAATCGGAG |
| YTHDF2 | AGCCCCACTTCCTACCAGATG | TGAGAACTGTTATTTCCCCATGC |
| YTHDF3 | TCAGAGTAACAGCTATCCACCA | GGTTGTCAGATATGGCATAGGCT |
| HNRNPC | TCCTCCTCCTATTGCTCGGG | GTGTTTCCTGATACACGCTGA |
| HNRNPA2B1 | ATTGATGGGAGAGTAGTTGAGCC | AATTCCGCCAACAAACAGCTT |
| FTO | ACTTGGCTCCCTTATCTGACC | TGTGCAGTGTGAGAAAGGCTT |
| ALKBH5 | CGGCGAAGGCTACACTTACG | CCACCAGCTTTTGGATCACCA |
| NOP2 | AAGGGTGCCGAGACAGAACT | GAGCACGACTAGACAGCCTC |
| NSUN2 | CAAGCTGTTCGAGCACTACTAC | CTCCCTGAGAGCGTCCATGA |
| NSUN3 | CATGCTGGCAATATGCTGTCC | AAAGATCCCTGAGAGAGTGTGT |
| NSUN4 | CCATCAATCCGTGTCAGTCTC | GCTTAGCACTTACATGATCCCAG |
| NSUN5 | CGCTACCATGAGGTCCACTAC | GCATCTCGCACCACGTCTT |
| NSUN6 | TTAAGAGGAGCCCATGTCTATGC | CTTTGCGGCTTAGTTCAGAAATC |
| NSUN7 | GGACTCCGTTTATGTCATGGC | CTCAGACTCGGACAAGGACC |
| DNMT1 | AGGCGGCTCAAAGATTTGGAA | GCAGAAATTCGTGCAAGAGATTC |
| DNMT3A | CCGATGCTGGGGACAAGAAT | CCCGTCATCCACCAAGACAC |
| DNMT3B | AGGGAAGACTCGATCCTCGTC | GTGTGTAGCTTAGCAGACTGG |
| TRDMT1 | CGGGTGCTGGAGCTATACAG | CGACAGTGTTGACATCAATGGC |
| TET2 | GATAGAACCAACCATGTTGAGGG | TGGAGCTTTGTAGCCAGAGGT |
| TET3 | GCCGGTCAATGGTGCTAGAG | CGGTTGAAGGTTTCATAGAGCC |
| ALYREF | GCAGGCCAAAACAACTTCCC | AGTTCCTGAATATCGGCGTCT |
| YBX1 | GGGGACAAGAAGGTCATCGC | CGAAGGTACTTCCTGGGGTTA |
